# Di-HAMP domains of a cytoplasmic chemoreceptor modulate nucleoid array formation and downstream signaling

**DOI:** 10.1128/mbio.00057-25

**Published:** 2025-04-18

**Authors:** P. J. Jazleena, Apurba Das, Annick Guiseppi, Fabian Debard, Jaya Sharma, Mutum Yaikhomba, Tâm Mignot, Emilia M. F. Mauriello, Pananghat Gayathri

**Affiliations:** 1 Biology, Indian Institute of Science Education and Research Pune, Pashan, Pune, India; 2 Laboratoire de Chimie Bactérienne, CNRS, Aix-Marseille Univ 128791, Marseille, Provence-Alpes-Côte d'Azur, France; Institut Pasteur, Paris, France

**Keywords:** MCP, HAMP domain, FrzCD, motility, signal transduction, *Myxococcus xanthus*

## Abstract

In bacterial chemosensing, environmental cues are typically sensed by bacterial transmembrane receptors known as methyl-accepting chemotaxis proteins (MCPs). MCPs form highly organized arrays using the bacterial membrane as a scaffold. These arrays amplify the signals and transduce them into a cellular response. The FrzCD cytoplasmic receptor from *Myxococcus xanthus* is unique due to its ability to bind DNA and use the nucleoid as a scaffold to form arrays. In this study, we identified two HAMP (histidine kinase, adenylyl cyclase, MCP, and phosphatase) domains located between the DNA binding and signaling domains of FrzCD. *In vitro* experiments demonstrate that the di-HAMP domain restricts FrzCD to a dimeric form in solution and modulate FrzCD affinity for DNA, whereas the signaling domain stabilizes higher-order oligomeric assemblies upon DNA binding. Through fluorescence microscopy and analyses of *M. xanthus* social behavior, we demonstrate that the impact of the FrzCD HAMP domains on DNA binding and oligomerization significantly influences the formation of Frz clusters on the nucleoid as well as group motility and development. Our results suggest that the di-HAMP domain might have roles not only in signal transduction but also in the plasticity of chemosensory arrays. These observations illustrate mechanisms of regulation of a DNA-bound cytoplasmic array formed by a diffusible MCP.

**IMPORTANCE:**

Our study identifies the presence of a di-HAMP domain in a cytoplasmic chemoreceptor, FrzCD, from *Myxococcus xanthus*, and highlights its role in dynamic receptor oligomerization on a DNA scaffold. By controlling receptor oligomerization and subsequently the array formation on the nucleoid, the di-HAMP domain imparts plasticity to receptor arrays. Such plasticity governs cellular responses to external signals and dictates bacterial social behaviors such as group motility and multicellular structure formation.

## INTRODUCTION

Bacteria sense external signals and convey them into various cellular behaviors through intricate regulatory pathways known as chemosensory systems (CSS). Chemosensory systems regulate diverse behavioral responses, including taxis, gene expression, and development (1–3). The chemical signal is sensed by specialized chemoreceptors, referred to as methyl-accepting chemotaxis proteins (MCPs). A canonical MCP comprises a periplasmic ligand-binding domain, followed by a transmembrane region, a HAMP (histidine kinase, adenylyl cyclase, MCP, and phosphatase) domain, and a methyl-accepting (MA) signaling domain in its C-terminal cytoplasmic region (2). The HAMP domain is a homodimeric four-helix bundle where each monomer is ~50 amino acids (4). It comprises two amphipathic helices, AS1 and AS2, joined by an approximately 14-residue flexible loop as a connector (4–6). Structural studies revealed two different HAMP conformations with different helical registers, rotation, and crossing angles that dictate opposite downstream signals in bacterial chemotaxis (7). The helices in the HAMP domain undergo relative movements when the MCP is bound to its ligand, as observed from the structural states of the NarQ receptor captured with and without the ligand nitrate (8). Beyond signal transduction, HAMP domains are also shown to control the cellular localization of receptors in *Pseudomonas aeruginosa* (9).

A common feature of MCPs is their ability to oligomerize and form highly ordered hexagonal arrays, where each core complex is composed of two trimers of dimers of MCP, two CheW docking proteins, and one CheA dimer (10–12). In these arrays, MCP hexagons, consisting of six MCP trimers of dimers, are networked by CheA–CheW rings essential for the integrity of the system (13, 14). Receptor arrays are essential for the amplification of the initial signal, which is a direct consequence of the cooperative interactions between clustered chemoreceptors (13, 15, 16). While arrays have been described as universal among prokaryotic and archaeal chemoreceptors (17), their subcellular localization and distribution vary among different bacterial species. Membrane chemosensory arrays are the most common and can be visualized by fluorescence microscopy as clusters located at the cell poles (18). However, there are MCPs that lack the transmembrane domain and are located in the cytoplasm (18, 19). FrzCD, the MCP of the Frz system of the gliding bacterium *Myxococcus xanthus*, is an example of a cytoplasmic MCP (20, 21).

**Fig 1:**
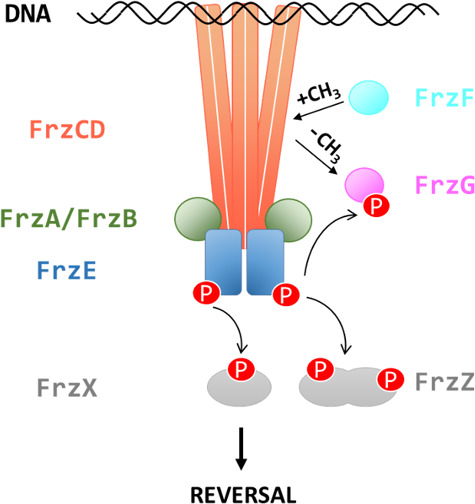
The *M. xanthus* Frz chemosensory pathway**.** The chemoreceptor FrzCD interacts with the adapter proteins FrzA/FrzB and the histidine kinase FrzE to form clusters on the nucleoid. FrzX and FrzZ are CheY proteins. FrzF and FrzG are the methyltransferase and the methylesterase, respectively, that dictate the methylation status of FrzCD. The pathway regulates the frequency at which bacteria reverse their movement direction.

*M. xanthus* is a bacterium with a multistage developmental cycle that involves swarming, predation, and aggregation into specialized multicellular biofilms termed fruiting bodies. Out of eight CSS and a total of 21 chemoreceptors in *M. xanthus* (22), the most studied CSS is the Frz system, which modulates the frequency at which cells reverse their direction of movement in a chemotactic-like manner (23). This regulation is critical for a successful developmental cycle because the aggregation of *M. xanthus* cells into fruiting bodies is dependent on the frequency of reversals (24). In addition to FrzCD, the cytoplasmic MCP, the Frz system comprises FrzE (homologous to CheA); FrzA and FrzB (homologous to CheW); two CheY-like response regulators, FrzX and FrzZ; as well as FrzF and FrzG (homologous to CheR and CheB, respectively) (Fig. 1) (24, 25). How environmental signals are perceived by FrzCD remains elusive as this protein lacks an obvious sensing domain.

**Fig 2:**
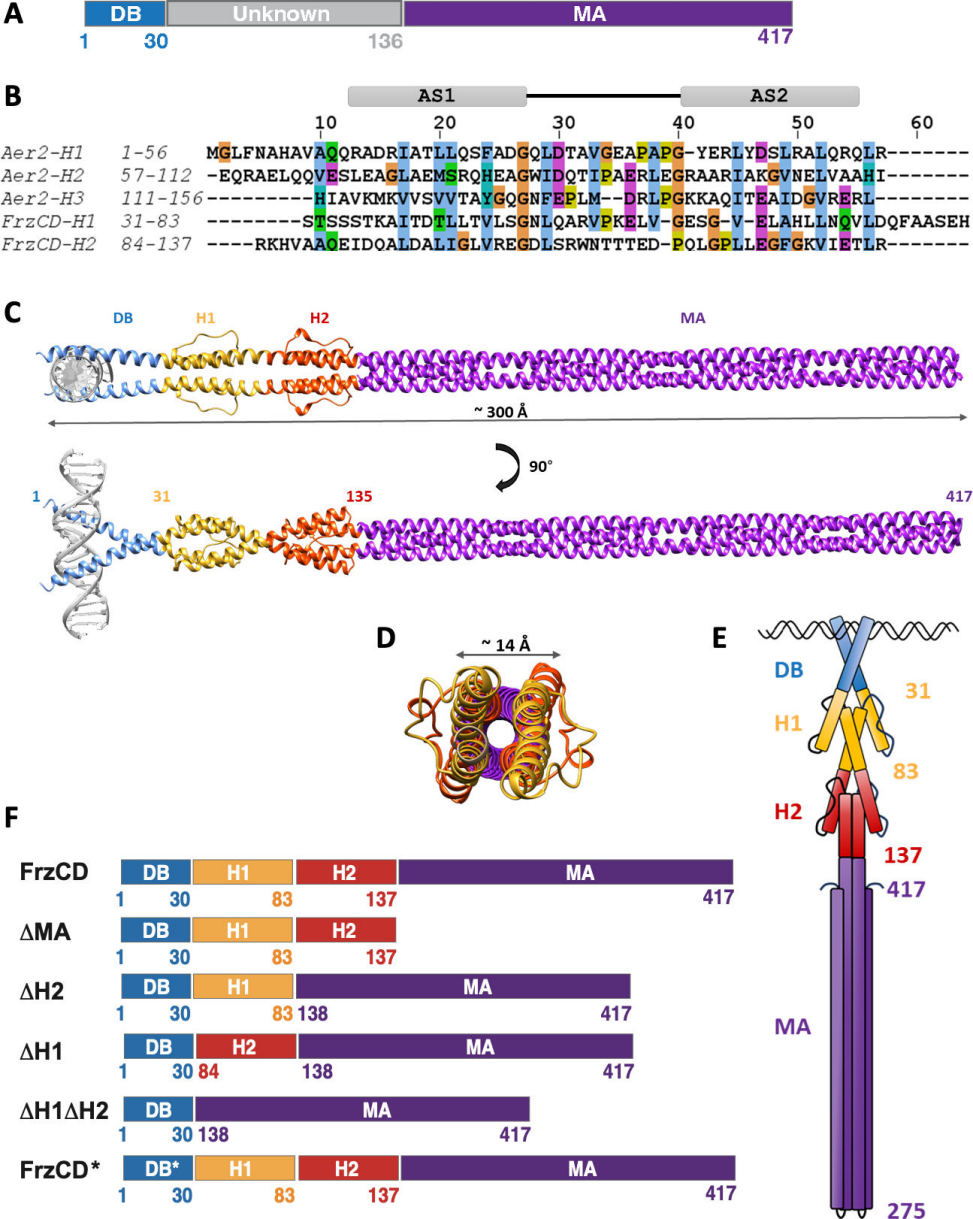
FrzCD possesses two HAMP domains between the DB and MA domains. (**A**) FrzCD comprises an N-terminal DNA-binding (DB) domain (1–30) and a C-terminal methyl-accepting (MA) domain (137–417). Residues 31 to 136 are of unknown fold, as inferred from sequence-based predictions. (**B**) Sequence alignment of residues 31 to 83 and 84 to 136 of FrzCD with the HAMP domains of the tri-HAMP region from the Aer2 receptor, shows the presence of two alpha helices (AS1 and AS2) in each region. We thus renamed regions 31 to 83 and 84 to 136 of FrzCD, HAMP1 (H1) and HAMP2 (H2). (**C**) Predictions suggest that the FrzCD dimer has an elongated structure of about 300 Å (see also Fig. S2E). The domain architecture of FrzCD comprises an N-terminal DNA-binding region (DB: residues 1 to 30), followed by a di-HAMP unit composed of HAMP1 (H1: residues 31 to 83) and HAMP2 (H2: residues 84 to 137) terminating in a long C-terminal coiled-coil signaling domain (MA: residues 138 to 417). (**D**) Schematic representation end-on views of the dimeric FrzCD. An end-on view of the dimeric model shows that the loops connecting the AS1 and AS2 helices of H1 and H2 project out from the cylindrical surface. The MA domain subtends a circular view of approximately 14 Å diameter. (**E**) Schematic representation of a FrzCD dimer, as depicted from our structural predictions. AS1 of HAMP1 is continuous with the DB domain, and AS2 of HAMP1 is connected to AS1 of HAMP2 by a short helical linker. AS2 of HAMP2 is continuous with the helix of the MA coiled-coil domain. (**F**) Schematic representation of various FrzCD domain deletions.

Besides a conserved C-terminal methyl-accepting (MA) domain (residues 137 to 417), FrzCD features a positively charged N-terminal amino acid stretch (residues 1 to 30) responsible for DNA-binding (DB domain) (Fig. 2A) (21). The function and fold of residues 31 to 136 are unknown. We have previously shown that FrzCD localizes into multiple clusters distributed along the nucleoid (20, 21, 26). Colocalization of FrzCD with the nucleoid is due to the direct interaction of the FrzCD N-terminal domain with DNA, in a DNA sequence-independent manner (21). Removal of the N-terminal region of FrzCD causes the loss of association with the nucleoid and the dispersal of FrzCD in the cytoplasm, indicating that the binding of FrzCD to DNA is required for cluster formation (21). We also showed that the outcome of this nucleoid-mediated clustering of FrzCD is the same as that of transmembrane chemosensory arrays, which is the ability of the system to respond cooperatively to external signals (21).

In this study, we identified the existence of a di-HAMP domain between the DNA-binding and the methyl-accepting coiled coil domain of FrzCD. We show that the absence of one or both HAMP domains leads to aberrant Frz clustering on DNA in single cells and altered reversal frequencies with motility and developmental defects as consequences. Our complementary *in vitro* data suggest that while the MA domain allows FrzCD oligomerization, the di-HAMP domain restricts the oligomeric status of FrzCD in solution to a dimer. This suggests that the di-HAMP domain might prevent cluster formation when FrzCD is free in the cytoplasm and before it encounters the nucleoid. Thus, HAMP domains play a crucial role in regulating DNA binding, cluster formation on the nucleoid, exposure of methylated residues in the MA domain, and thereby reversal frequencies. In summary, this study provides insights into the array formation of a cytoplasmic MCP, very few of which are characterized to date, and how DNA binding and array formation are modulated by the protein domain architecture.

## RESULTS

### Structure prediction and homology show the presence of a di-HAMP domain in FrzCD

Secondary structure predictions indicated that the N-terminal portion of FrzCD, from residue 31 to 136, forms alpha helices interrupted by two nonhelical gaps (residues 49–61 and 106–118) (Fig. S1A and B). This observation led us to examine the sequence for the presence of multiple HAMP domains. While common domain prediction servers, such as Superfamily, CD-VIST, or SMART (27–29), could not identify HAMP domains in FrzCD, a visual inspection, followed by sequence alignment with other poly-HAMP domains, revealed two consecutive HAMP domains, labeled as H1 (residue 31 to 83) and H2 (residue 84 to 137) (Fig. S1B). The alignment also showed the presence of conserved glycine residues flanking the loop region (Fig. S1B). Similar to H1 and H2 from the tri-HAMP region of the Aer2 receptor from *P. aeruginosa*, H1 and H2 from FrzCD are connected by a continuous helical linker, as opposed to the concatenated H2 and H3 of Aer2 (Fig. 2B) (4).

A homology model of FrzCD showed a cylindrical structure with a length of approximately 300 Å and a diameter of approximately 14 Å (Fig. 2C through E). The AS1 helix of H1 immediately follows the DB domain of FrzCD, and the AS2 of H2 precedes the MA domain. Apart from the disordered DB domain, the structure predicted by AlphaFold2 aligned well with our homology model for FrzCD (Fig. S2A and B). H1 and H2 from FrzCD might also adopt different conformations alike H1 and H2 from Aer2 (PDB: 3LNR (4)). The predominant alpha helical content was validated by circular dichroism spectroscopy (Fig. S2C).

These results allowed us to redefine the FrzCD domain architecture, which includes now a newly identified di-HAMP unit comprising H1 (residues 31 to 83) and H2 (residues 84 to 136), positioned between the DB (bZIP-like) and the MA domains (Fig. 2E; Fig. S2D and E). We proceeded to validate our model experimentally using domain-wise deletion constructs (Fig. 2F).

### HAMP domains of FrzCD modulate the methylation-mediated regulation of reversal frequency

To probe the role of FrzCD HAMP domains in protein activity and protein localization at the nucleoid *in vivo*, we first constructed a FrzCD-mNeongreen (FrzCDmNG) fusion. Similar to a previous FrzCD-GFP construct (20–22, 26), FrzCDmNG exhibited stable expression and formed clusters at the nucleoid when expressed at the endogenous locus and under the *frz* promoter but was not fully functional in mediating swarming behavior (Fig. S3A through C). However, when the same fusion was expressed under a promoter inducible by vanillate at a heterologous locus (*P_van_-frzCDmNG*), it fully complemented the absence of the native copy of *frzCD* (Fig. S3C). The compromised functionality of FrzCDmNG when expressed at the endogenous locus was due to a 7.84 ± 0.96 log_10_-fold increase in the expression (as measured by q-RT PCR) of the co-transcribed downstream gene, *frzE*, compared to the wild type. Finally, the ability of *P_van_-frzCDmNG* to form clusters was indistinguishable from that observed for *frzCDmNG* at the endogenous locus (Fig. S3B).

**Fig 3:**
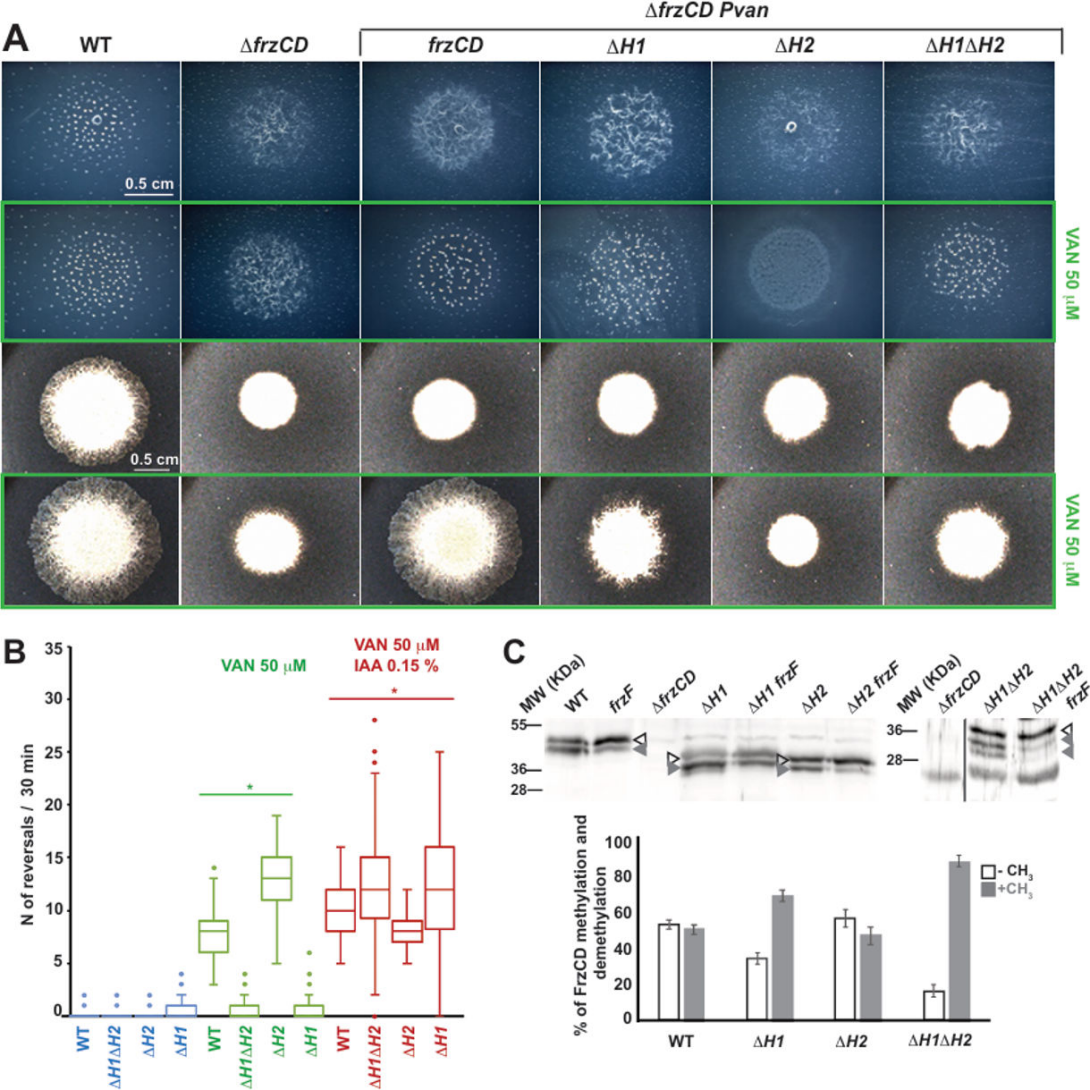
FrzCD HAMP domains are important for the methylation-mediated regulation of reversal frequency and motility. (**A**) Representative phenotypic assays of DZ2 (wild type), EM525 (Δ*frzCD*), EM885 (Δ*frzCD P_van_-frzCDmNG*), EM914 (Δ*frzCD P_van_-frzCDmNG*^Δ*H1*^), EM913 (Δ*frzCD P_van_-frzCDmNG*^Δ*H2*^), and EM911 (Δ*frzCD P_van_-frzCDmNG*^Δ*H1*Δ*H2*^). The first two rows are fruiting body formation assays on 1.5% CF agar, in the presence (green rectangle) or absence of 50 µM vanillate, imaged at 72 hours. The last two rows are motility phenotypes on 0.5% CYE agar, in the presence (green rectangle) or absence of 50 µM vanillate, imaged at 48 hours. (**B**) Box plots of reversal frequencies of EM885 (Δ*frzCD P_van_-frzCDmNG*), EM914 (Δ*frzCD P_van_-frzCDmNG*^Δ*H1*^), EM913 (Δ*frzCD P_van_-frzCDmNG*^Δ*H2*^), or EM911 (Δ*frzCD P_van_-frzCDmNG*^Δ*H1*Δ*H2*^) single cells moving on agar pads supplemented or not with 50 µM vanillate and 0.15% IAA. The lower and upper boundaries of the boxes correspond to 25th and 75th percentiles, respectively. The median is shown as a line at the center of each box, and circles represent the 10th and 90th percentiles. For the reversal frequency measurements, 100 cells from two biological replicates were used. (**C**) Representative Western blot on whole-cell extracts of strains DZ2 (wild type), EM525 (Δ*frzCD*), EM775 (*frzCD*^Δ*H2*^), EM776 (*frzCD*^Δ*H1*Δ*H2*^), EM889 (*frzCD*^Δ*H1*^), EM1083 (*frzF*), EM1084 (*frzF frzCD*^Δ*H1*^), EM1085 (*frzF frzCD*^Δ*H2*^), and EM1086 (*frzF frzCD*^Δ*H1*Δ*H2*^) showing the methylated (gray arrowheads) and demethylated (white arrowheads) forms of FrzCD. The histogram shows the average percentage of intensity of methylated and demethylated bands on the total FrzCD intensity amount. Results are issued from three independent biological replicates. The error bars correspond to standard deviations.

Next, we systematically deleted either or both of the HAMP domains from the *P_van_-frzCDmNG* construct (Fig. 2F) and induced the various alleles in a Δ*frzCD* strain. The deletion of either H1 or H2 resulted in distinct phenotypes, indicating that these two domains are functionally nonredundant. Single-cell motility behaviors suggest that the motility defect observed in the absence of H1 could be attributed to decreased single-cell reversal frequencies, measured to be as low as those of Δ*frzCD* cells (Fig. 3A and B). On the other hand, the motility defects of cells lacking H2 were due to increased reversal frequencies, reaching approximately twice that of the wild type (Fig. 3A and B). Additionally, while reduced reversals did not affect fruiting body formation in *P_van_-frzCDmNG*^Δ*H1*^, increased reversals prevented the formation of fruiting bodies in *P_van_-frzCDmNG*^Δ*H2*^ (Fig. 3A and B), similar to other strains with increased reversals (24). *P_van_-frzCDmNG*^Δ*H1*Δ*H2*^ phenocopied *P_van_-frzCDmNG*^Δ*H1*^ in reversal frequencies and fruiting body formation (Fig. 3A and B).

The ability of cells to modulate their reversal frequencies is contingent on the methylation state of FrzCD, wherein low methylation levels correlate with high reversal frequencies and, conversely, high methylation with fewer reversals (30). Therefore, we sought to check whether the reversal frequency defects of our FrzCD HAMP mutants were also correlated with their methylation states. Given the difficulties in resolving methylation in FrzCDmNG by Western blot due to its high molecular weight, we generated in-frame deletions of each or both HAMP domains at the endogenous locus and performed methylation assays. FrzCD is methylated by FrzF (CheR homolog) on glutamate or glutamine residues forming glutamyl-methylesters, with the glutamine residues subject to post-translational deamidation by FrzG (CheB homolog) before methylation (31). Previous work shows that the fully unmethylated form of FrzCD migrates slowly and corresponds to the upper band in Western blot analyses, and the lower bands correspond to a heterogeneous population of methylated and deamidated species (32, 33) (Fig. 3C).

To better discriminate the methylated/deamidated from the nonmethylated forms, we systematically knocked-out the gene encoding the methyltransferase *frzF* from each strain. The comparison of the protein profiles shows that while methylation/deamidation levels of FrzCD^ΔH1^ were higher than those of the wild type, for FrzCD^ΔH2,^ demethylation was prevalent (Fig. 3C). Furthermore, for FrzCD^ΔH1ΔH2^ the methylation/deamidation levels were similar to those of FrzCD^ΔH1^. Interestingly, the deamidase activity of FrzG seems particularly pronounced in FrzCD^ΔH1^, as even in the absence of FrzF, the faster band is intense (Fig. 3C). These results showed that the absence of the HAMP domains altered the methylation of FrzCD, with further downstream effects.

The simultaneous deletion of H1 and H2 resulted in phenotypes similar to those of *P_van_-frzCDmNG*^Δ*H1*^, implying a dominant effect of Δ*H1* on Δ*H2* (Fig. 3A through C). Notably, *P_van_-frzCDmNG*^Δ*H1*^, *P_van_-frzCDmNG,*^Δ*H2*^ and *P_van_-frzCDmNG*^Δ*H1*Δ*H2*^ single cells all responded to a known activator of FrzCD, the isoamyl alcohol (IAA) (34), by frequently reversing their movement direction, similar to wild type (Fig. 3B). This suggests that all the above protein variants were able to signal to downstream Frz proteins in the presence of high signal levels. The phenotypes of HAMP mutants were not due to expression defects of the *frzCD* alleles (Fig. S3D). Together, these results suggest that the absence of the HAMP domains altered the FrzCD activity, but in the presence of high signal concentrations, the ability of the FrzCD variants to transduce signals remained unchanged compared to the wild type.

### FrzCD HAMP domain deletions have differential effects on cluster formation upon nucleoid binding

**Fig 4:**
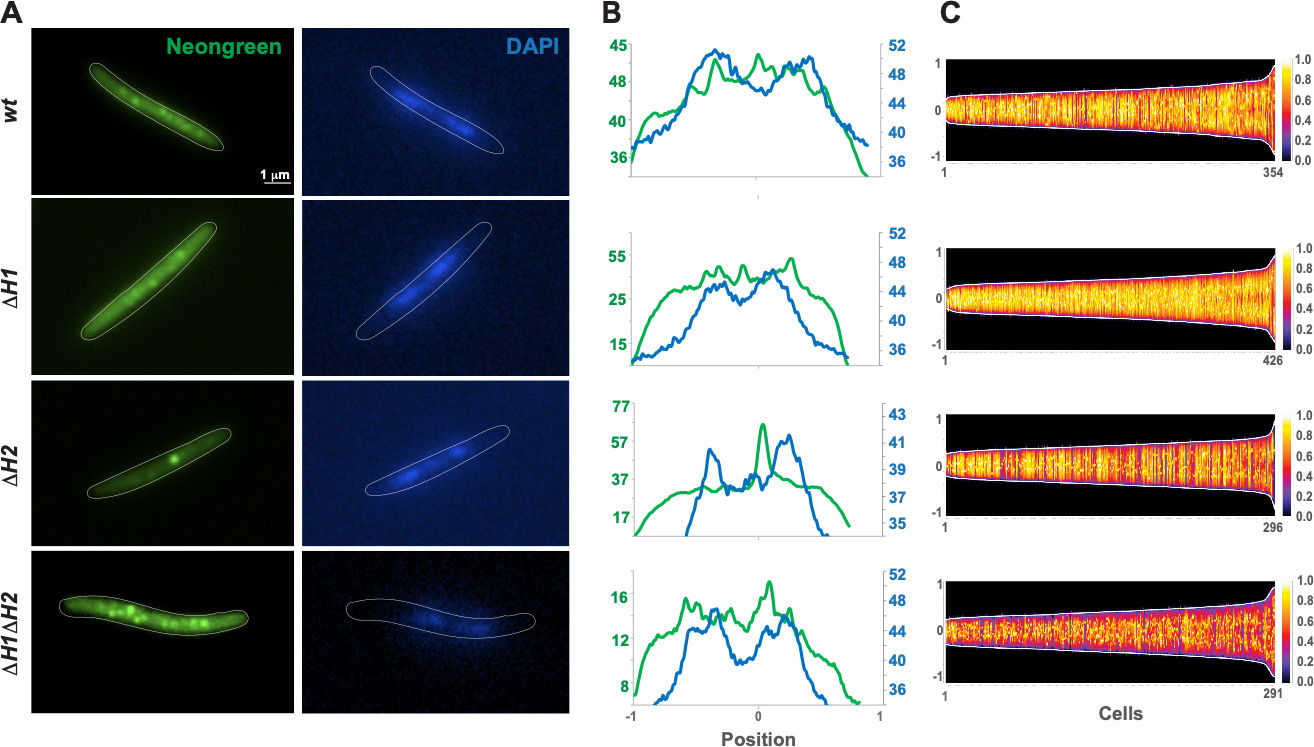
FrzCD HAMP domains play a role in cluster formation on the nucleoid. (**A**) Representative micrographs of *M. xanthus* cells from strains EM885 (Δ*frzCD P_van_-frzCDmNG*), EM914 (Δ*frzCD P_van_-frzCDmNG*^Δ*H1*^), EM913 (Δ*frzCD P_van_-frzCDmNG*^Δ*H2*^), or EM911 (Δ*frzCD P_van_-frzCDmNG*^Δ*H1*Δ*H2*^) labeled with Neongreen and DNA stained with the DAPI stain. The genetic backgrounds of the *M. xanthus* strains are indicated on the left. Scale bars correspond to 1 µm. (**B**) Neongreen (green) and DAPI (blue) fluorescence profiles with the fluorescence intensity (arbitrary units) represented on the *y*-axis and the cell length positions, with −1 and +1 indicating the poles and 0 the center, on the *x*-axis. (**C**) For each indicated strain, at least 290 cells (*x*-axis) from at least two biological replicates are represented as lines and ordered according to their length (pixels) in demographs. The Neongreen fluorescence intensity along the cell body is represented as colored pixels at the corresponding cell position (from −1 to +1 on the *y*-axis). “0” is the cell center. On the right, a scale indicates the fluorescence intensity and the corresponding colors.

To establish a correlation between the role of HAMP domains in the FrzCD activity (Fig. 3) and FrzCD localization to the bacterial nucleoid, we looked at the cellular localization of different FrzCD HAMP mutants fused to Neongreen. Similar to the previously described FrzCD-GFP (21), FrzCD-mNG formed multiple clusters that were evenly distributed on the nucleoid (Fig. 4; Fig. S4A). Deletions of each or both HAMP domains did not impede either cluster formation or DNA binding as *M. xanthus* cells bearing these deletions all formed Frz clusters at the nucleoid (Fig. 4; Fig. S4A). However, the functions of H1 and H2 in cluster formation were not redundant as cells with either deletion exhibited clusters with different localization patterns. In the absence of H1, clusters resembled those formed by FrzCD-mNG in intensity and distribution (Fig. 4; Fig. S4), although they appeared less defined (Fig. 4C). In the absence of H2, cells formed fewer clusters but with high fluorescence intensities (Fig. 4; Fig. S4). Finally, the deletion of both HAMP domains resulted in the formation of highly condensed clusters (Fig. 4), slightly more numerous than those in the wild type (Fig. S4). The fluorescence intensity of FrzCD^ΔH1ΔH2^-mNG clusters was similar to that of FrzCD-mNG, but cells showed less diffused fluorescence, suggesting that all FrzCD^ΔH1ΔH2^-mNG protein units are engaged in clusters (Fig. 4). In conclusion, HAMP domains were not necessary for cluster formation, but their absence appeared to impact the cluster intensity and number.

### HAMP domains reduce the affinity of FrzCD for DNA, while the MA domain favors FrzCD–DNA stable complexes

The observed changes in cluster formation among the various HAMP mutants could be due to impaired DNA-binding. To estimate the affinity of FrzCD for DNA in the presence or absence of its HAMP domains, we heterologously expressed and purified various His_6_-tagged FrzCD domains, as well as the full-length protein. The purification yielded stable and soluble proteins, supporting a reliable prediction of domain boundaries (Fig. S5).

As shown previously, the interaction of FrzCD with DNA is charge-dependent and independent of the DNA sequence, length (from 69 to 1,254 bp), and GC content (21). We compared the DNA binding of HAMP deletion constructs of FrzCD with the wild type. By performing electromobility shift assays (EMSA), we confirmed that full-length FrzCD binds to DNA fragments as short as 8 bp and up to 600 bp (Fig. S6 and S7A). FrzCD also bound to 8-base single-stranded DNA fragments (Fig. S7B), strengthening the hypothesis that the FrzCD–DNA interaction is predominantly charge-based and involves the DNA phosphate backbone. To provide direct evidence for this charge-mediated interaction, we replaced three lysine (K9, K13, and K18) and two arginine (R15 and R17) residues of FrzCD with negatively charged glutamates (FrzCD*). The DNA-binding capacity of this FrzCD* variant was completely abolished (Fig. S7C), confirming that the FrzCD–DNA interaction is predominantly charge-mediated.

FrzCD^ΔMA^ exhibited stable binding comparable to that of the wild type only to very short DNA fragments of 8 bp to 12 bp (Fig. S6). Smearing was observed for DNA fragments longer than 12 bp, indicative of unstable complex formation. While 8 bp is probably the minimal footprint of a dimeric helical structure (35), larger DNA fragments might require a stable FrzCD oligomerization for the DNA–FrzCD complex formation. The MA domain might thus be important for the stabilization of FrzCD oligomers on large DNA fragments (Fig. S6 and S7A). A comparison of the binding profiles of the FrzCD* variant with that of FrzCD^ΔMA^ (Fig. S7C) strongly suggests that the smears observed in FrzCD^ΔMA^ does not indicate a complete abrogation of DNA binding, but rather the formation of an unstable complex.

**Fig 5:**
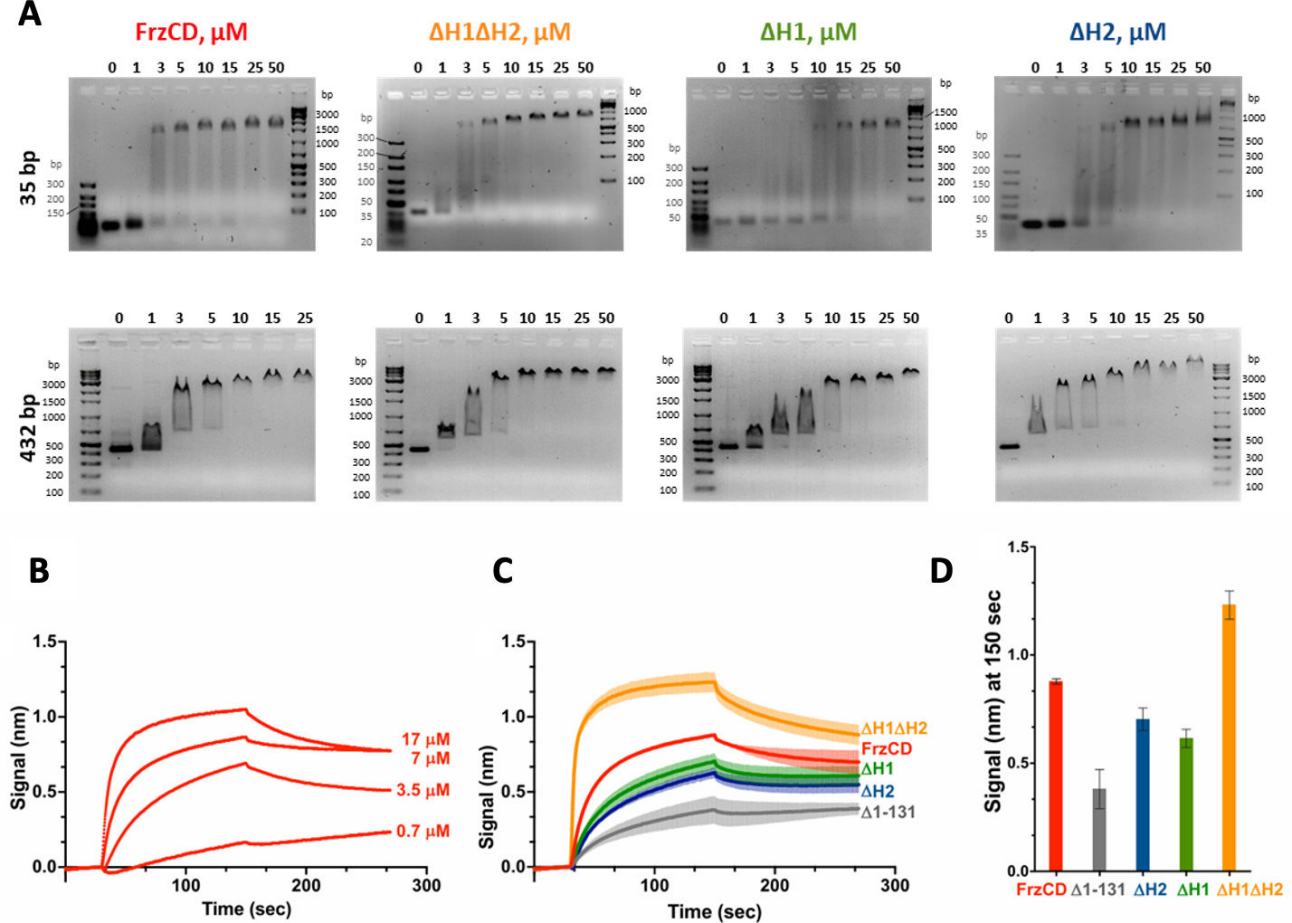
HAMP domain deletion constructs of FrzCD have different affinities for DNA. (**A**) Representative electrophoretic mobility shift assays (EMSA) resolving the binding to DNA. The indicated concentrations of purified FrzCD protein domains as well as FrzCD full-length were incubated with 0.5 µM DNA fragments of the indicated sizes. DNA ladders are indicated as base pairs. (**B**) Binding curves of increasing concentration of His_6_-FrzCD with an immobilized 178 bp DNA fragment at a concentration of 5 nM. (**C**) Average binding curves and biological replicates in color gradations of the indicated His_6_-FrzCD variants at 7 µM concentration, with an immobilized 178 bp DNA fragment at a concentration of 5 nM. (**D**) Average binding signals at 150 seconds, extrapolated from panel **C** for the indicated FrzCD variants. Error bars indicate standard deviations for two biological replicates. The sequences of the DNA fragments used in panels A–D are tabulated in Table S4.

Next, we compared the affinity of FrzCD for DNA in the presence or absence of its HAMP domains. Stable shifts were observed for FrzCD constructs lacking one or both HAMP domains (FrzCD^ΔH2^, FrzCD^ΔH1,^ or FrzCD^ΔH1ΔH2^) with DNA fragments from 35 to 432 bp (Fig. 5A). This supported the observation that all these FrzCD variants mediated cluster formation on the nucleoid (Fig. 4). Interestingly, there seemed to be a difference in the DNA-binding affinity for FrzCD constructs lacking one or both HAMP domains (Fig. 5A).

To quantify FrzCD–DNA binding and explore the dynamics of FrzCD binding to DNA, we used Bio-layer Interferometry (BLI), as previously described (21). First, we optimized the binding of FrzCD to the biosensor by testing different FrzCD concentrations (Fig. 5B). We then compared the DNA-binding of FrzCD mutants lacking the HAMP domains with that of FrzCD full-length at 7 µM. In line with the gel-shift experiments, FrzCD^ΔH1ΔH2^ showed a higher affinity for DNA than the wild type (0.21 ± 0.02 µM vs 0.65 ± 0.6 µM; number after “±” denotes standard deviation [SD]) (Fig. 5C and D). FrzCD^ΔH1^ and FrzCD^ΔH2^ exhibited slightly decreased affinities for DNA compared to the wild type (Fig. 5C and D), with *K_D_* values of 1.61 ± 1.08 µM and 1.73 ± 1.29 µM, respectively (numbers after “±” denote SD). As expected, very low DNA binding was detected when FrzCD^Δ1-131^ was used (Fig. 5C and D). Together with gel shift results and microscopy observations, we consider the FrzCD^Δ1-131^ residual binding to DNA detected by BLI to be a nonspecific effect. Finally, while the deletion of either H1 and H2 decreases the affinity of FrzCD for DNA, their simultaneous absence significantly favors FrzCD binding to DNA.

### Di-HAMP domains restrict oligomerization in the absence of DNA

**Fig 6:**
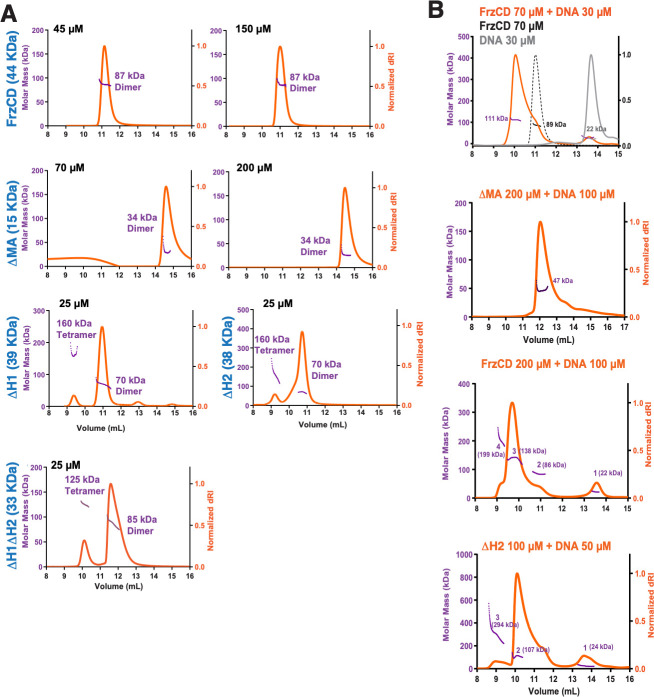
FrzCD exhibits a tendency to oligomerize in the absence of its di-HAMP domain. (**A**) SEC-MALS profiles for the indicated forms of FrzCD. Proteins were used at the indicated concentrations. The axes read as in panel B. (**B**) SEC-MALS profiles for the indicated forms of FrzCD in the presence of a 35 bp DNA. The gray line in the first plot represents the expected elution of DNA alone, and the black dotted line represents the expected elution of dimeric FrzCD. Proteins and DNA were used at the indicated concentrations, and the orange line represents the elution of the indicated forms of FrzCD in the presence of DNA. The y-axes represent molar mass (left, purple) and normalized differential refractive index (dRI; orange), while the x-axis denotes the volume of elution. The molecular masses corresponding to each peak are labeled. Table S5 summarizes the calculated molecular weights based on the SEC-MALS experiments and the expected molecular weights of the protein–DNA complexes.

The notion that transmembrane MCPs form high-ordered structures, coupled with the observation of FrzCD–DNA complexes of increasing molecular weights (supershifts) in EMSA, suggests the formation of FrzCD oligomers on DNA. Moreover, supershifts are also evident in the absence of the MA and HAMP domains (Fig. 5; Fig. S6 and S7A). Hence, we decided to test the oligomerization of our different FrzCD constructs in the absence and presence of DNA. Given that FrzCD has an elongated shape and is not globular, we employed size exclusion chromatography combined with multi-angle light scattering (SEC-MALS) measurements to accurately estimate the oligomeric status. Results indicate that full-length FrzCD and FrzCD^ΔMA^ exist as dimers even at input concentrations as high as 150 and 200 µM, respectively (Fig. 6A). Conversely, FrzCD^ΔH1^, FrzCD,^ΔH2^ or FrzCD^ΔH1ΔH2^ form higher oligomers, showing the presence of tetramers even at lower protein concentrations (Fig. 6A). These observations suggest that the absence of either H1, H2, or both HAMP domains favors a nonnative oligomeric state *in vitro*.

To investigate whether the inability of FrzCD^ΔMA^ to form stable complexes with high-molecular-weight DNA was due to an oligomerization defect, we decided to characterize the oligomerization status of both FrzCD and FrzCD^ΔMA^ upon DNA binding. For the SEC-MALS experiments, a 35 bp oligonucleotide was used so that the elution profiles of free DNA and free protein were well-resolved (Fig. 6B). Upon addition of DNA, both FrzCD and FrzCD^ΔMA^ eluted as complexes with DNA, but, most strikingly, FrzCD formed high-order oligomers at high protein concentration (Fig. 6B), unlike in the absence of DNA (Fig. 6A). On the other hand, FrzCD^ΔMA^ could only be resolved as a dimer, even in complex with DNA and at high protein concentrations (Fig. 6B). This observation is consistent with the EMSA results, where supershifts were not observed for FrzCD^ΔMA^ (Fig. S6). Lastly, FrzCD^ΔH2^ also formed high-order oligomers in the presence of DNA (Fig. 6B), as in its absence (Fig. 6A). Table S5 summarizes the calculated molecular weights based on the SEC-MALS experiments and the expected molecular weights of the protein–DNA complexes. Together, these results indicate that while the MA domain is required for FrzCD oligomerization, the di-HAMP domains might prevent such oligomerization when DNA is absent.

## DISCUSSION

MCP array formation is an essential feature for the allosteric activation of the chemosensory pathway and the generation of amplified responses for functioning over a wide range of signal concentrations (13). In the absence of a transmembrane domain, the cytoplasmic MCP FrzCD utilizes the nucleoid as a scaffold to form dense, dynamic clusters (21, 26, 36). The dynamic nature of these clusters likely stems from the intrinsic plasticity of FrzCD arrays (26), resulting from a delicate equilibrium between oligomerization and molecular turnover among arrays. In this work, we report the presence of a di-HAMP domain connecting the DB and MA signaling domains of FrzCD. The existence of an HAMP domain in FrzCD was proposed in an earlier study (37). However, the exact boundaries of this putative HAMP domain, roughly aligning with our H2 domain, remained imprecise due to the absence of detailed structure predictions (37). Here, through a combination of structure predictions and careful visual sequence comparison, we identified two sequential HAMP domains, H1 and H2, connected by a short helical linker. These domains exhibit conserved nonhelical loops that extend out of the elongated and predominantly helical structure of FrzCD, a typical feature of the HAMP domain architecture.

**Fig 7:**
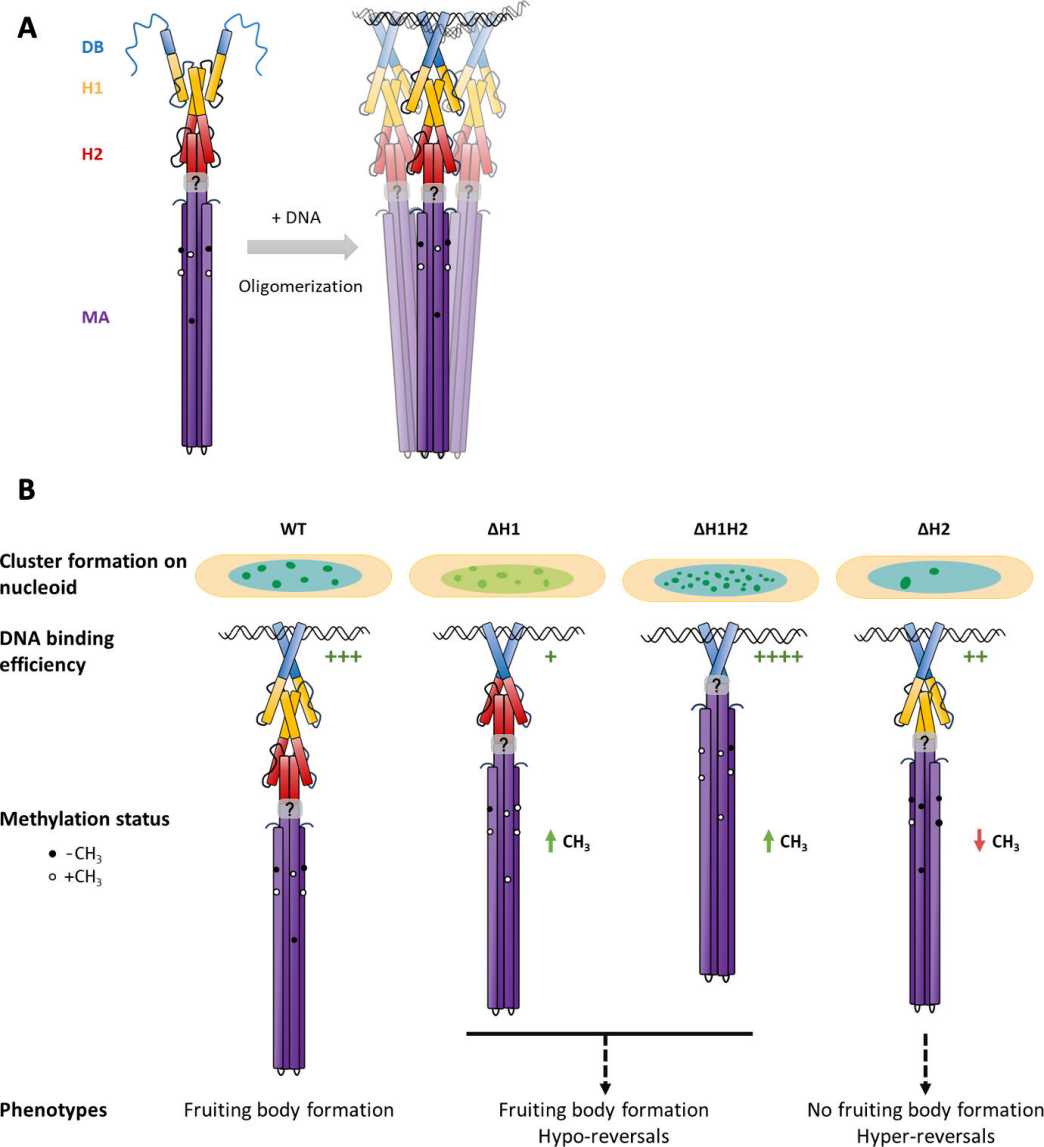
Model for the regulation of oligomerization and signaling by HAMP domains. Abbreviations and color codes read as in Fig.1. (**A**) DNA-binding by the positively charged N-terminal region of the FrzCD DB domain induces conformational changes that are transduced by the di-HAMP domain to the MA domain. This conformational change might reorient the MA helices to favor oligomer formation possibly by axial rotation, allowing the exposure of an interface responsible for oligomerization. (**B**) Alterations in FrzCD oligomerization and its affinity for DNA, due to the absence of one or both HAMP domains, significantly impact FrzCD cluster formation on the nucleoid. When both HAMP domains H1 and H2 are deleted, the affinity of FrzCD for DNA increases substantially *in vitro*, which likely accounts for the formation of well-defined and bright, though functionally impaired, nucleoid clusters. Additionally, while the deletion of either H1 or H2 facilitates oligomerization in the absence of DNA, the absence of either HAMP domain reduces the affinity for DNA. This observation might explain the more diffuse clusters seen in FrzCD^ΔH1^ and the reduced number of clusters in FrzCD^ΔH2^ compared to the wild type. The proposed rotation of the HAMP domains, which is transmitted to the methyl-accepting (MA) domain, may affect the accessibility of the methylation sites on the MA domain. Consequently, impaired methylation states lead to defects in reversal frequencies and social behaviors.

Based on *in vitro* experiments, we show that the presence of the di-HAMP domain primarily prevents the formation of higher-order oligomers of FrzCD in the absence of DNA. This could explain the oligomerization of the nonnative variant of FrzCD lacking the di-HAMP domain and imply that a probable function of the di-HAMP is to restrict the FrzCD oligomerization to a dimer in the absence of DNA. We also show that the DNA-dependent oligomerization beyond the dimeric state strictly depends on the presence of the MA domain. This regulation might be crucial to prevent higher-order oligomerization when freely diffusing FrzCD molecules have not encountered the nucleoid yet in the bacterial cytoplasm. A probable molecular basis through which the di-HAMP domain restricts the oligomerization state of FrzCD to a dimer in solution can be explained by the following model. Following the initial DNA-binding primarily via the positively charged residues on the FrzCD DB domain, a conformational change might induce the axial rotation of the HAMP helices and, in turn, a reorientation of the MA helices that favors the formation of trimers of dimers (Fig. 7A). These conformational changes may also lead to a differential exposure of methylatable residues in the MA domain. The presence of two HAMP domains also influences the DNA-binding ability of FrzCD. Indeed, the simultaneous deletion of H1 and H2 significantly enhances the affinity of FrzCD for DNA *in vitro*. This behavior might lead to the formation of well-defined and intense, albeit not fully functional, nucleoid-FrzCDmNG^ΔH1ΔH2^ clusters *in vivo* (Fig. 7B). Conversely, the reduced affinity of FrzCD^ΔH1^ and FrzCD^ΔH2^ for DNA might explain the reduced cluster intensity and number, respectively, of these allelic variants.

The proposed rotation of the FrzCD HAMP domains is consistent with the reported rotation of AS1 and AS2, the molecular mechanism by which HAMP domains mediate the transduction of signals from the sensing to the MA domain of chemoreceptors (5). Our model is also coherent with what is reported for other poly-HAMP domains (7). In these poly-HAMP domains, molecular movements including helix rotation, translation, or tilt facilitate conformational transitions between HAMP conformers that propagate opposing downstream signals. The specific arrangement of two HAMP domains in FrzCD might have evolved to ensure signal transduction when the membrane scaffold is replaced by DNA. A better understanding of the sensing mechanisms of FrzCD in the future should further explain the requirement of two HAMP domains. The interplay between DNA-binding events and consequent structural dynamics contributes to the regulation of FrzCD oligomerization states in response to its cellular environment.

The rotation model that we propose might also explain the methylation and phenotypic defects observed in the HAMP deletion strains (Fig. 7B). The di-HAMP domain might allow to alternate conformations similar to H1 and H2 of the Aer2 tri-HAMP region, where H1 and H2 conformers result in alternate chemosensory signals. According to our model, the putative rotation of the HAMP domains, transduced to the MA domain, might influence the accessibility of the methylation sites on the MA domain. Impaired methylation states result, in turn, in alterations in reversal frequencies and social behaviors. However, a high signal concentration overrides the impaired methylation states.

Two additional interesting results emerged from our *in vitro* study. First, the dispensability of the MA domain in the formation of FrzCD dimers implies that the DB and HAMP domains alone are sufficient for FrzCD dimerization. Interestingly, our EMSA assays show that both FrzCD and FrzCD^ΔMA^ can efficiently form complexes with DNA fragments as short as 8 bp. This DNA length corresponds to the minimal footprint of a dimeric helical structure (35), suggesting that the DB domain could bind DNA in a dimeric form. The higher-order oligomerization of the MA domain suggests that in the presence of the MA domain, FrzCD can form oligomers *in vitro* even without its downstream Frz partners. However, *in vivo,* clusters of FrzCD were observed very rarely in the absence of FrzA and FrzE (21, 26). This suggests that other factors are likely required for efficient array formation and its stability *in vivo*, by modulating the HAMP domains and thereby the relative orientation of the helices of the MA domain. A better understanding of the sensing mechanisms of FrzCD in the future will likely contribute to further explaining the requirement of two HAMP domains.

In conclusion, this study brings new insights into the role of HAMP domains, not only in signal transduction but also in the plasticity of chemosensory arrays, established by a delicate equilibrium between oligomerization and molecule turnover. The presence of two consecutive HAMP domains emerges as an intrinsic necessity when scaffolds other than the inner membrane are utilized to nucleate receptor arrays. This introduces a novel dimension to the understanding of these universally conserved domains, revealing their potential significance beyond their conventional role in signal transduction.

## MATERIALS AND METHODS

### Domain architecture prediction for FrzCD

The secondary structure of FrzCD was predicted using Psipred (38) and Jpred 4 (39) (Fig. S1A). Further, servers such as Superfamily (27), CD-VIST (28), or SMART (29) were employed to determine the domains of FrzCD. A multiple sequence alignment (MSA) was performed for the alignment of FrzCD against poly-HAMP domains of other MCPs, such as Aer2 and HAMP consensus sequences obtained from the works by Dunin–Horkawicz and Lupas (40) using Jalview software (Fig. S1B) (41). The alignment was inspected for the presence of HAMP domains. Based on the secondary structure prediction as well as MSA, domain boundaries were predicted for FrzCD (Fig. 2E and F).

### Bioinformatics analysis and modeling of FrzCD

FrzCD was dissected into the DNA-binding (DB), di-HAMP (H1 and H2), and methyl-accepting (MA) domains. The N-terminal DNA-binding domain sequence was aligned against various protein structures from basic leucine zipper (bZIP) families such as 1GU4, Creb1, ATF4, Fos, and Jun using Jalview software (Fig. S2D). To obtain a homology model for FrzCD, the approach included modeling each of these domains separately as dimers with a 12-residue overlap to put the model together. Therefore, for DB, 1–42 residues were modeled against GCN4 (PDB: 2dgc), while the di-HAMP domain (residues 31–136) were modeled against HAMP1 and HAMP2 of Aer2 (PDB: 3lnr) using SWISS-MODEL (42). The four-helix bundle of the MA dimer (residues 124–417) was generated using CCBuilder (43). Dimers of the three domains were superimposed based on C-α overlap regions. Each domain was individually modeled with a 12-residue overlap (equivalent to three alpha-helical turns), and subsequently, superimposition using C⍺ atoms (Fig. S2E), allowing to create an entire FrzCD dimer model. The domains were joined at residues Ser34 and Ser35 between DB and H1 and residues Val130 and Ile131 between H2 and MA to generate the FrzCD homology model. Recently, a dimer model of FrzCD was also generated in AlphaFold2 using ColabFold (Fig. S2B) (44, 45).

### Bacterial strains and plasmids

The strains and plasmids used in this work are listed in Table S1 and S2, respectively. *M. xanthus* strains were grown in the casitone yeast extract (CYE) reach medium or in the developmental casitone fruiting (CF) medium. For all experiments, *M. xanthus* cells were resuspended in Tris-phosphate-magnesium medium (TPM). To create *M. xanthus* in-frame deletion strains, 900 bp upstream and downstream of the region targeted for deletion were amplified by PCR and cloned into the pBJ114 vector (24) previously digested with HindIII and EcoRI. To fuse FrzCD to the Neongreen reporter, *neongreen* was amplified and cloned into pDPA20 (20), previously digested with NotI and XhoI, to replace *gfp*. To generate *M. xanthus* strains expressing *frzCD-neongreen* alleles at the endogenous locus, *frzCD* variants plus an upstream region for a total of 900 bp, *neongreen* and 900 bp downstream of *frzCD* on the *M. xanthus* chromosome were amplified and cloned into pBJ114 previously digested with HindIII and EcoRI. To obtain the plasmids for the inducible expression, the different *frzCD-neongreen* alleles were amplified from each respective *M. xanthus* strain and cloned into pMR3690 (46) previously digested with NdeI and BglII. *M. xanthus-*competent cells were prepared and transformed by electroporation, as described previously (24). Cells were grown in CYE-rich medium containing, when required, kanamycin at a final concentration of 25 µg/mL. Vanillate was used to induce the expression of the different *frzCD-neongreen* alleles at a concentration ranging from 0.5 to 50 µM.

*Escherichia coli* DH-5α (New England Biolabs) and *E. coli* BL21-AI (Invitrogen) were used as the cloning and expression hosts, respectively, while pHis17 (refer to Addgene plasmid #78201 for the vector backbone, obtained from Löwe lab, MRC LMB, Cambridge, UK) and pETphos (47) were the plasmid vectors employed for the heterologous and inducible overexpression, respectively. Cells were grown at 37°C in the LB medium containing ampicillin at a final concentration of 100 µg/mL.

For cloning in pHis17, the full-length *frzCD* or DNA coding for *frzCD* variants were amplified with primers containing the *NdeI* and *BamHI* restriction sites and the sequence to insert a hexa-histidine tag at the protein C terminus. The primers as listed in Table S3 were used for domain deletions using a restriction-free cloning strategy and site-directed mutagenesis (48). For cloning in pETphos, *frzCD* variants were amplified and cloned into pETphos previously digested with NdeI and BamHI. Thus, we obtained ΔMA, ΔH1, ΔH2, ΔH1H2, and FrzCD* (a mutant where three lysines K9, K13, and K18 and two arginines R15 and R17 in the DB region were replaced by glutamates) constructs. Positive clones were confirmed by sequencing, and *E. coli* expression strains such as BL21-AI, BL21-DE3, and C43 (BL21-DE3 derivative) were transformed with plasmids for overexpression.

### Motility phenotypes

For motility phenotypic assays, exponentially growing cells in the CYE medium at 32°C were adjusted to an OD_600_ of 10 in TPM buffer and spotted (5 µL) on CYE plates containing an agar concentration of 0.5% or on CF plates containing an agar concentration of 1.5%. The plates without or with vanillate at concentrations ranging from 0.5 to 50 µM were incubated at 32°C and photographed after 48 hours (for twitching motility) or 72 hours (for fruiting body formation) with an Olympus SZ61 binocular.

### Reversal frequencies

Two microliters of cells from 4 × 10^8^ cfu mL^−1^ vegetative CYE cultures was spotted on a thin fresh TPM agar supplied or not with 0.15% IAA. Time-lapse movies were recorded for 1 hour with frames captured every 30 seconds. Movies were obtained and reversal frequencies manually counted. Reversal frequencies were measured from cells issued from two biological replicates. Box plots were generated by R, and statistical significance was obtained with a Wilcoxon test from the R software (https://www.r-project.org/).

### Fluorescence microscopy and image analysis

For fluorescence microscopy analyses, 5 µL of cells from 4 × 10^8^ cfu mL^−1^ vegetative CYE cultures was spotted on a thin fresh TPM agar pad at the top of a slide. A cover slip was added immediately on the top of the pad, and the obtained slide was analyzed by microscopy using a Nikon TE PFS inverted epifluorescence microscope (100× oil objective, NA 1.3, phase contrast).

To study the colocalization with DNA, the TPM agar pads were supplied with 1 µg/mL DAPI stain and 50 mM vanillate. Prior to imaging, *M. xanthus* cells were grown in 50 mM vanillate. Cell fluorescence profiles were obtained with the “plot profile” function of FIJI. FrzCD cluster position and intensity were represented as demographics and histograms automatically generated with the “MicrobeJ” Fiji/ImageJ plugin created by A. Ducret, Brun Lab (https://brunlab.com/research/microbej/). All data plots and statistical tests were obtained with the R software (https://www.r-project.org/). Results are issued from biological triplicates.

### Western and methylation analysis

Total cell lysates were analyzed with sodium dodecyl sulfate-polyacrylamide gel electrophoresis (SDS-PAGE) and transferred to a nitrocellulose membrane. After transfer, the membrane was blocked for 2 hours at room temperature in 5% powdered milk. Proteins were detected using αFrzCD (1:10,000) (18) from rabbit in a mixture with Tris-buffered saline with Tween 20 (OriGene Technologies Inc., Rockville, MD, USA) in combination with a secondary anti-rabbit antibody (from goat) coupled to horseradish peroxidase (HRP) (1:5,000) (Thermo Fisher Scientific, MA, USA). Methylation was measured by FIJI as the percentage of the intensity of the upper band over the intensity of all bands for each given sample. Demethylation was extrapolated as the total intensity minus the intensity from the methylated band.

### Protein purification

Proteins expressed from the pHis17 plasmid were purified by affinity chromatography (HisTrap, GE Life Sciences) and ion-exchange chromatography at 4°C. *E. coli* protein expression strains such as BL21-AI, BL21-DE3, and C43 (BL21-DE3 derivative), standardized for expression for each construct, were grown in LB broth supplemented with 100 µg/mL ampicillin at 37°C until OD_600_ reached 0.6. Expression was induced with 0.2% arabinose or 0.5 mM IPTG, and cells were incubated at 30°C for 5 hours or 18°C for 10 hours. Cells were harvested and resuspended in lysis buffer (200 mM NaCl, 50 mM Tris [pH 8], 10% glycerol) and sonicated at 60% amplitude (three cycles of 1 s on, 3s off for 2 minutes each, with 5 minute intervals). The lysate was centrifuged at 35,000 × *g* for 45 minutes at 4°C, and the supernatant was loaded onto a 5 mL HisTrap FF column pre-equilibrated with buffer A200 (200 mM NaCl, 50 mM Tris, pH 8.0). Washes with buffer A200 and 2% and 5% buffer B200 (200 mM NaCl, 500 mM imidazole, 50 mM Tris [pH 8.0], mixed with A200) were carried out before elution with a step gradient of 10%, 20%, 50%, and 100% of buffer B200 (mixed with A200). EDTA was added to the 5 mL eluted fractions (final concentration of EDTA, 1 mM) to prevent protein degradation. Pure protein fractions identified from SDS-PAGE were pooled and dialyzed against A50 (50 mM NaCl, 1 mM EDTA, 50 mM Tris [pH 8.0]). Ion-exchange chromatography using MonoQ/MonoS 10/100 Gl (GE Healthcare) was then performed to remove impurities, including bound DNA. Proteins were loaded with A50 buffer and eluted using a NaCl gradient from 50 mM to 335 mM (0% to 30% A1000, 1000 mM NaCl, 1 mM EDTA, 50 mM Tris, pH 8.0) over 20 column volumes. Eluted protein fractions were concentrated with centricons (Vivaspin 10 kDa or 3 kDa MWCO), aliquoted, flash-frozen, and stored at −80°C for experiments in Fig. 4A and 5; Fig. S6 and S7. Purified proteins separated by SDS-PAGE are shown in Fig. S5A.

Proteins expressed from pETphos were purified by affinity chromatography (HisTrap, GE Life Sciences). For protein purification, strains for each construct were grown in LB broth supplemented with 100 µg/mL ampicillin and incubated at 37°C until the OD_600_ reached 0.6. Protein expression was induced by 0.5 mM IPTG, and cells were incubated at 18°C for 10 hours. Cells were harvested and resuspended in lysis buffer (300 mM NaCl, 50 mM Tris, pH 7.4), followed by two French press cycles at 1,000 psi. The lysate was spun at 18,000 × *g* for 20 minutes at 4°C, and the supernatant was loaded onto a 5 mL column containing 1 mL of agarose beads pre-equilibrated with Ni^2+^ and lysis buffer. The column was washed extensively with lysis buffer containing 10 mM and then 75 mM imidazole. Elution was carried out with lysis buffer containing 200 mM or 500 mM imidazole. Aliquots were subjected to SDS-PAGE, and the fractions containing pure protein were pooled and dialyzed against a buffer containing 150 mM NaCl and 10 mM Tris, pH 7.4. Fractions containing protein were concentrated using centricons (Vivaspin 3 kDa MWCO), aliquoted, flash-frozen, and stored at −80°C for further use in experiments shown in Fig. 5B. The SDS-PAGE profile of these proteins is shown in Fig. S5B.

### Circular dichroism spectroscopy

Far-UV CD measurements were carried out on a Jasco J-720 spectropolarimeter. The parameters used for measurements of far-UV CD spectra were as follows: step resolution, 1 nm; scan speed, 100 nm/minute; and bandwidth, 1 nm. The concentrations of FrzCD and DMA were 2.5 µM and 20 µM, respectively. CAPITO was used to analyze and plot CD data (49).

### Electrophoretic mobility shift assay (EMSA)

EMSAs were carried out for varying lengths of DNA including 8, 12, 20, 35, 69, 432, and 600 bp of which 432 and 600 bp were PCR amplicons, while the remaining DNA fragments were custom synthesized (Sigma and Eurofins) and annealed. A single-stranded (ss) poly A consisting of eight bases was also used, and all DNA sequences used for the study have been listed in Table S4. Increasing concentrations of purified proteins up to 50 µM were incubated with 0.5 to 2 µM of DNA at 25°C for 30 minutes in a reaction buffer comprising 50 mM NaCl, 1 mM dithiothreitol, 10% glycerol, and 50 mM, Tris pH 7.4. A higher concentration of DNA, 2 µM, was used for 8 bp DNA to allow visualization on an agarose gel. The samples were mixed with gel loading buffer and loaded on the appropriate percentage agarose gel containing ethidium bromide. For instance, 4% agarose gels were used for 8 bp, 12 bp, 20 bp, and 35 bp DNA, while 2% were used for 69 bp and 432 bp DNA. Samples with short DNA fragments were run at 80–90 V for 30 minutes in 1× TAE (40 mM Tris-acetate, 1 mM EDTA) buffer, followed by visualization under UV light. For longer DNA fragments, the gels were run at 100 V from 45 minutes to 1 hour. The gels were observed for a higher shift in the DNA bands in the presence of increasing protein concentrations.

### Biolayer interferometry

Protein–DNA interaction experiments were conducted at 25°C with the BLItz instrument from ForteBio (Menlo Park, CA, USA) as described previously (21, 26). A 178 bp DNA fragment was amplified with a forward primer conjugated at the 5′ with biotin (Eurogentec) and immobilized onto a streptavidin biosensor (ForteBio) in duplicate at a 5 nM concentration. Purified proteins were used as the analyte throughout the study at concentrations from 0.7 to 17 mM. The assay was conducted in 10 mM Tris, pH 7.4, and 150 mM NaCl. The binding reactions were performed with an initial baseline during 30 seconds, an association step at 120 seconds, and a dissociation step of 120 seconds, with lateral shaking at 2,200 rpm. A sensor reference subtraction was applied to account for nonspecific binding, background, and signal drift to minimize sensor variability.

### Size exclusion chromatography with multi-angle light scattering (SEC-MALS)

SEC coupled with multi-angle light scattering was employed to estimate the molar mass of FrzCD proteins in solution. Superdex 200 Increase 10/300 Gl (GE Healthcare) column coupled to Wyatt Dawn HELIOS II equipped with light scattering detectors at 18 angles along with a differential refractive index detector (Optilab TrEX) was used for the analysis. The column was equilibrated with buffer containing 50 mM NaCl and 50 mM Tris (pH 8.0) at room temperature (approximately 25°C), followed by injection of 100 µL of 45 µM and 150 µM (FrzCD); 70 µM and 200 µM (FrzCD^ΔMA^); 25 µM (FrzCD^ΔH1ΔH2^); 25 µM and 65 µM (FrzCD^ΔH2^); 25 µM (FrzCD^ΔH1^). All chromatograms were carried out at a flow rate of 0.4 mL/min. The observed molecular masses for proteins were estimated at various points along the peaks in elution curves, based on the differential refractive index and scattered intensities. The Zimm model of ASTRA software available with the equipment was used for fitting the data. Observed molecular masses were compared with the theoretical masses to estimate the oligomeric status of each protein. Bovine serum albumin (2 mg/mL) was used for calibration of the system.

Further, to determine a change in the oligomeric status of proteins upon DNA binding, chromatograms were carried out with the ligand, that is, 35 bp DNA was included in the injected samples. Hence, 100 µL of 70 µM FrzCD with 30 µM DNA; 200 µM FrzCD with 100 µM DNA; 200 µM FrzCD^ΔMA^ with 100 µM DNA; 100 µM FrzCD^ΔH2^ with 50 µM DNA were injected, while 30 µM DNA and 70 µM FrzCD served as controls. Observed molecular masses for all the peaks in the elution curve were estimated and compared with theoretical masses of putative protein–DNA species.

## AFTER EPUB

*[This article was published on 18 April 2025 with errors in equal-contribution statements. The statements were corrected in the current version, posted on 23 April 2025.]*

## SUPPLEMENTAL MATERIAL

Supplemental material mbio.00057-25-s0001.pdf Supplemental figures and tables.

ASM does not own the copyrights to Supplemental Material that may be linked to, or accessed through, an article. The authors have granted ASM a non-exclusive, world-wide license to publish the Supplemental Material files. Please contact the corresponding author directly for reuse.
