# Supplementary material for: Di-HAMP domains of a cytoplasmic chemoreceptor modulate nucleoid array formation and downstream signaling
Source: mBio. Author manuscript; Available in PMC 2025 May 15. (PMC12077210; doi:10.1128/mbio.00057-25)
Supplement: Supplementary [file EMS205020-supplement-Supplementary.pdf]

Supporting Information for

Di-HAMP domains of a cytoplasmic chemoreceptor modulate nucleoid array formation and downstream signalling

P J Jazleena[1†], Apurba Das[1†], Annick Guiseppi[2†], Fabien Debard[2‡], Jaya Sharma[1‡], Mutum Yaikhomba[1], Tâm Mignot[2], Emilia Mauriello[2*] and Pananghat Gayathri[1*]

[1] Biology, Indian Institute of Science Education and Research Pune, Dr. Homi Bhabha Road, Pashan, Pune, India 411008.
[2] Laboratoire de Chimie Bactérienne, CNRS, Aix-Marseille Univ, Marseille, France.
[†, ‡] These authors contributed equally to the work.
* To whom correspondence should be addressed: Emilia Mauriello, Pananghat Gayathri.

Email: emauriello@imm.cnrs.fr; gayathri@iiserpune.ac.in.

This PDF file includes:

Supporting text
Figures S1 to S7
Tables S1 to S5

Supplementary Figures

Figure S1

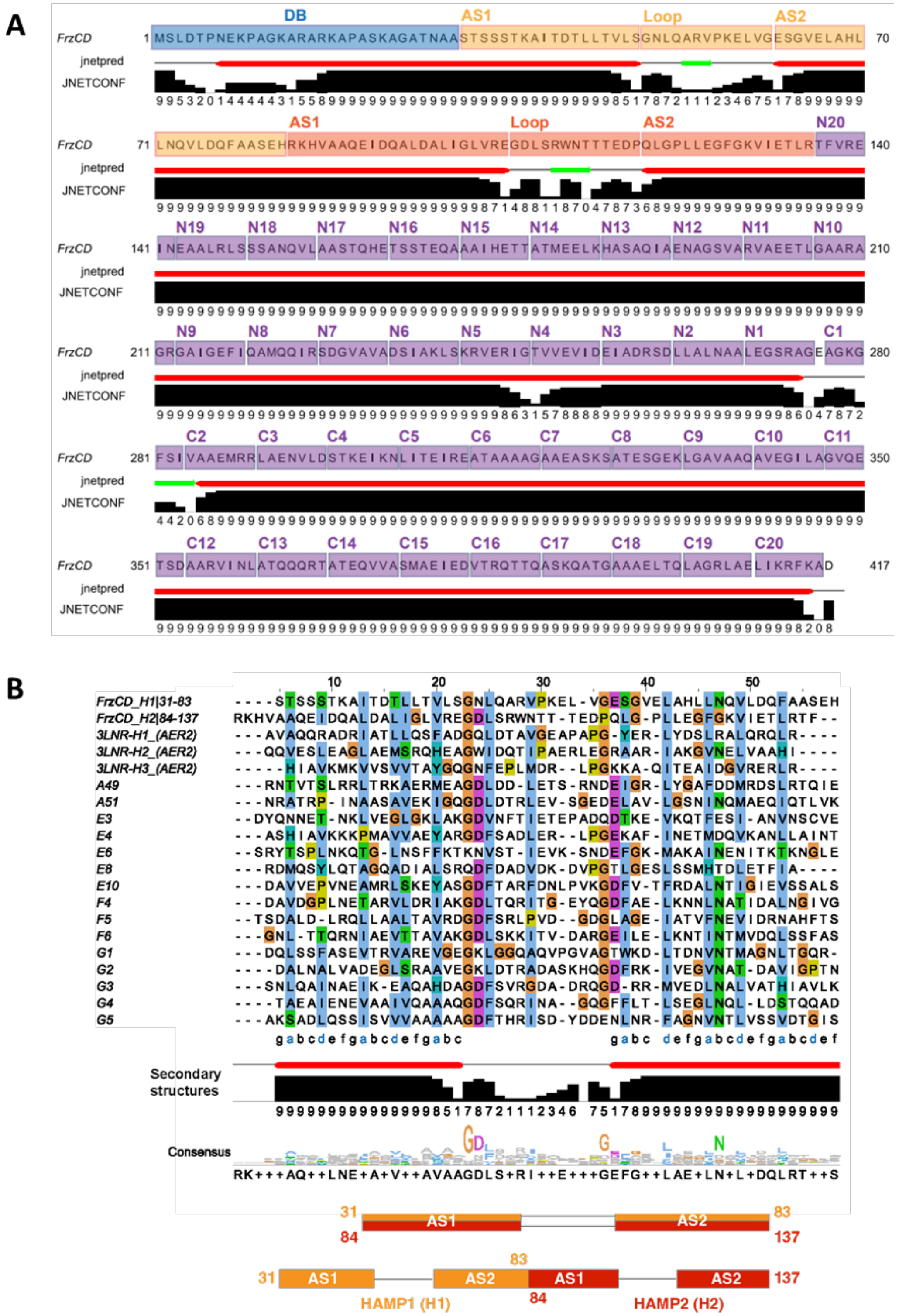

Supplementary Figure S1: FrzCD is mostly composed of alpha helices and its N-terminal domain comprises a di-HAMP domain.

A. Secondary structure prediction of FrzCD. DB (blue), H1 and H2 (each consisting of AS1, loop and AS2; orange and red), and MA (20 heptads of the N and C-terminal ends labeled as N1-20 and C1-20; purple) domains are marked on the amino acid sequence. Helices and strands are shown as red cylinders and green arrows, respectively. The confidence of secondary structure prediction is shown by numbers ranging from 0 to 9 (lowest to highest).

B. Sequence alignment (top) and secondary structure (bottom) suggest the presence of two concatenated HAMP domains in FrzCD (H1: residues 31-83 and H2: residues 84-137). The aligned sequences are the consensus sequences of various classes of HAMP domains (1). The color scheme, as per ClustalX, is used to represent the alignments. Hydrophobic residues of helices AS1 and AS2 of H1 and H2 are labeled in blue in the alignment. The conserved glycines flanking the loop region are in orange. AS2 of H1 is in tandem with AS1 of H2. The confidence of secondary structure prediction is shown by numbers ranging from 0 to 9 (lowest to highest).

Figure S2

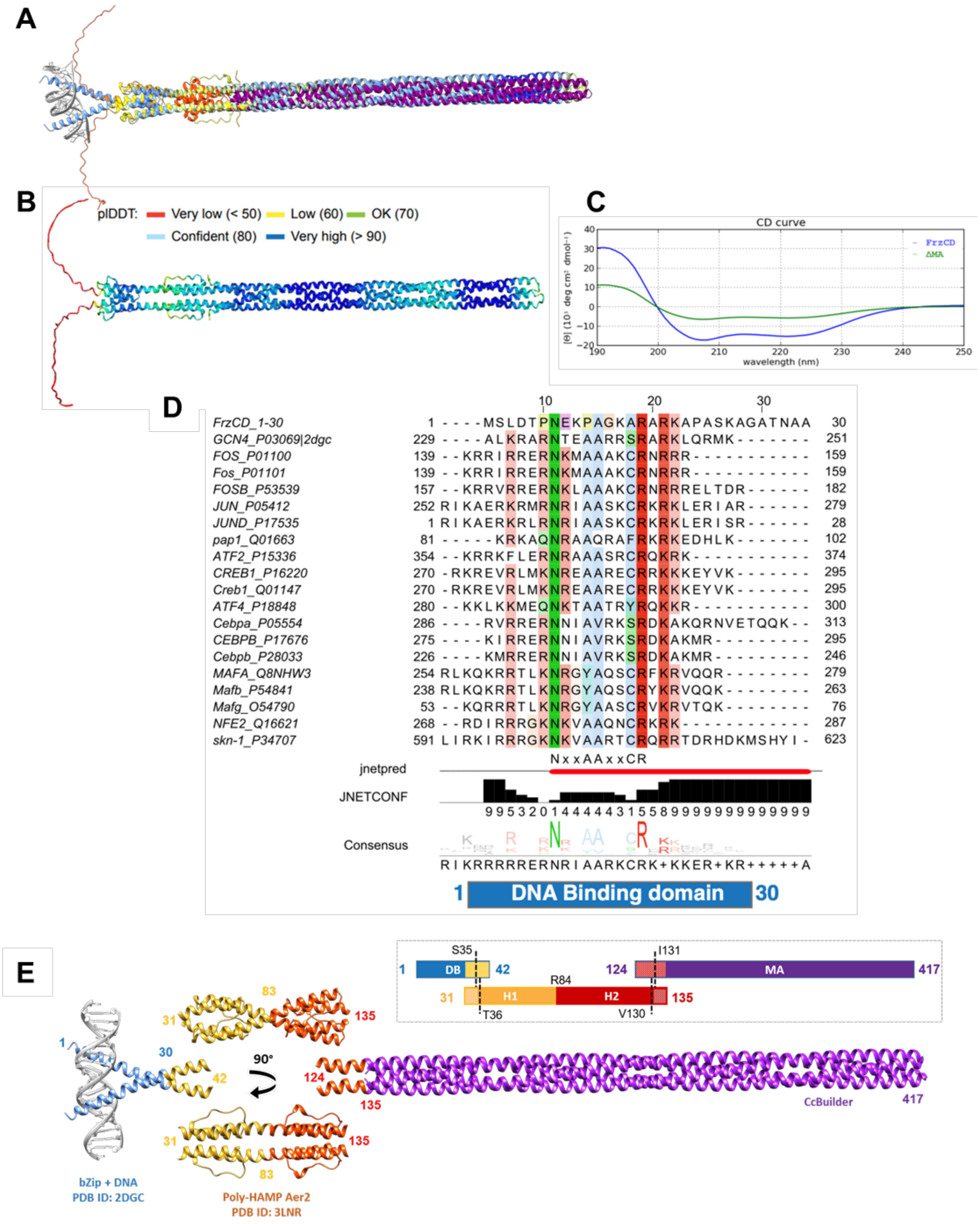
A
B
pIDDT: Very low (< 50) Low (60) OK (70)
Confident (80) Very high (> 90)
C
CD curve
FrzCD
ΔMA
[θ] (10³ deg cm² dmol⁻¹)
wavelength (nm)
D
10 20 30
FrzCD_1-30 1 ----MSLDTPNEKPAGKARARKAPASKAGATNAA 30
GCN4_P03069|2dgc 229 ----ALKRARNTEAARRSRARKLQRMK-------- 251
FOS_P01100 139 --KRRIRRERNKMAAAKCRNRRR----------- 159
Fos_P01101 139 --KRRIRRERNKMAAAKCRNRRR----------- 159
FOSB_P53539 157 --KRRVRRERNKLAAAKCRNRRRELTDR------ 182
JUN_P05412 252 RIKAERKRMRNRIAASKCRKRKLERIAR------ 279
JUND_P17535 1 RIKAERKRLRNRIAASKCRKRKLERISR------ 28
pap1_Q01663 81 -----KRKAQNRAAQRAFRKRKEDHLK------- 102
ATF2_P15336 354 --KRRKFLERNRAAASRCRQKRK----------- 374
CREB1_P16220 270 -RKREVRLMKNREAARECRRKKKEYVK------- 295
Creb1_Q01147 270 -RKREVRLMKNREAARECRRKKKEYVK------- 295
ATF4_P18848 280 --KKLKKMEQNKTAATRYRQKKR----------- 300
Cebpa_P05554 286 ----RVRRERNNIAVRKSRDKAKQRNVETQQK-- 313
CEBPB_P17676 275 ----KIRRERNNIAVRKSRDKAKMR--------- 295
Cebpb_P28033 226 ----KMRRERNNIAVRKSRDKAKMR--------- 246
MAFA_Q8NHW3 254 RLKQKRRTLKNRGYAQSCRFKRVQQR-------- 279
Mafb_P54841 238 RLKQKRRTLKNRGYAQSCRYKRVQQK-------- 263
Mafg_O54790 53 --KQRRRTLKNRGYAASCRVKRVTQK-------- 76
NFE2_Q16621 268 --RDIRRRGKNKVAAQNCRKRK------------ 287
skn-1_P34707 591 LIRKIRRRGKNKVAARTCRQRRTDRHDKMSHYI- 623
NxxAAxxCR
jnetpred
JNETCONF
9953201444443155899999999999999
Consensus
RIKRRRRRERNRIAARKCRK+KKER+KR+++++A
1 DNA Binding domain 30
E
S35
I131
1 DB 42
124 MA 417
R84
31 H1 H2 135
T36
V130
83
31
135
1
30
42
90°
124
135
CcBuilder
417
31
135
83
bZip + DNA
PDB ID: 2DGC
Poly-HAMP Aer2
PDB ID: 3LNR

Supplementary figure S2. Validation of homology model of FrzCD.

A. Superposition of the AlphaFold coordinates of FrzCD dimer mode (color-coded according to reliability scored by plDDT) with our homology model (color-coded according to domains; DB: blue, H1: golden, H2: red, MA: purple)

B. FrzCD AlphaFold model color-coded according to reliability scored by plDDT.

C. Circular dichroism spectroscopy of FrzCD (blue) and ΔMA (green) shows the presence of predominantly helical regions (71% and 44% helical content, respectively).

D. DNA binding domain of FrzCD is similar to bZIP DNA binding sequences. Sequence alignment of the DB domain with leucine zipper domains highlights the conserved consensus sequence NxxAAxxCR (where x is any residue) and positively charged residues (R and K). Secondary structure prediction of the DB domain highlights a helical conformation similar to basic leucine zipper proteins. The color scheme as per ClustalX, is used to represent the alignments.

E. Schematic representation of the strategy employed to generate a homology model of FrzCD where individual domains were modeled separately with an overlap of 12 amino acids for ease of stitching. The C-α of the 12 overlapping residues between each of the models were superimposed, followed by connecting S35 and T36 of DB and H1, and V130 and I131 of H2 and MA, respectively, and deleting the overlapping residue coordinates. Homology models for the DNA binding and di-HAMP domains were generated using leucine zipper (PDB ID: 2DGC) and Aer2 (PDB ID: 3LNR), respectively, in SWISS-MODEL while the MA domain was modeled using CCBuilder. The model is color-coded according to domains (DB: blue, H1: golden, H2: red, MA: purple).

Figure S3

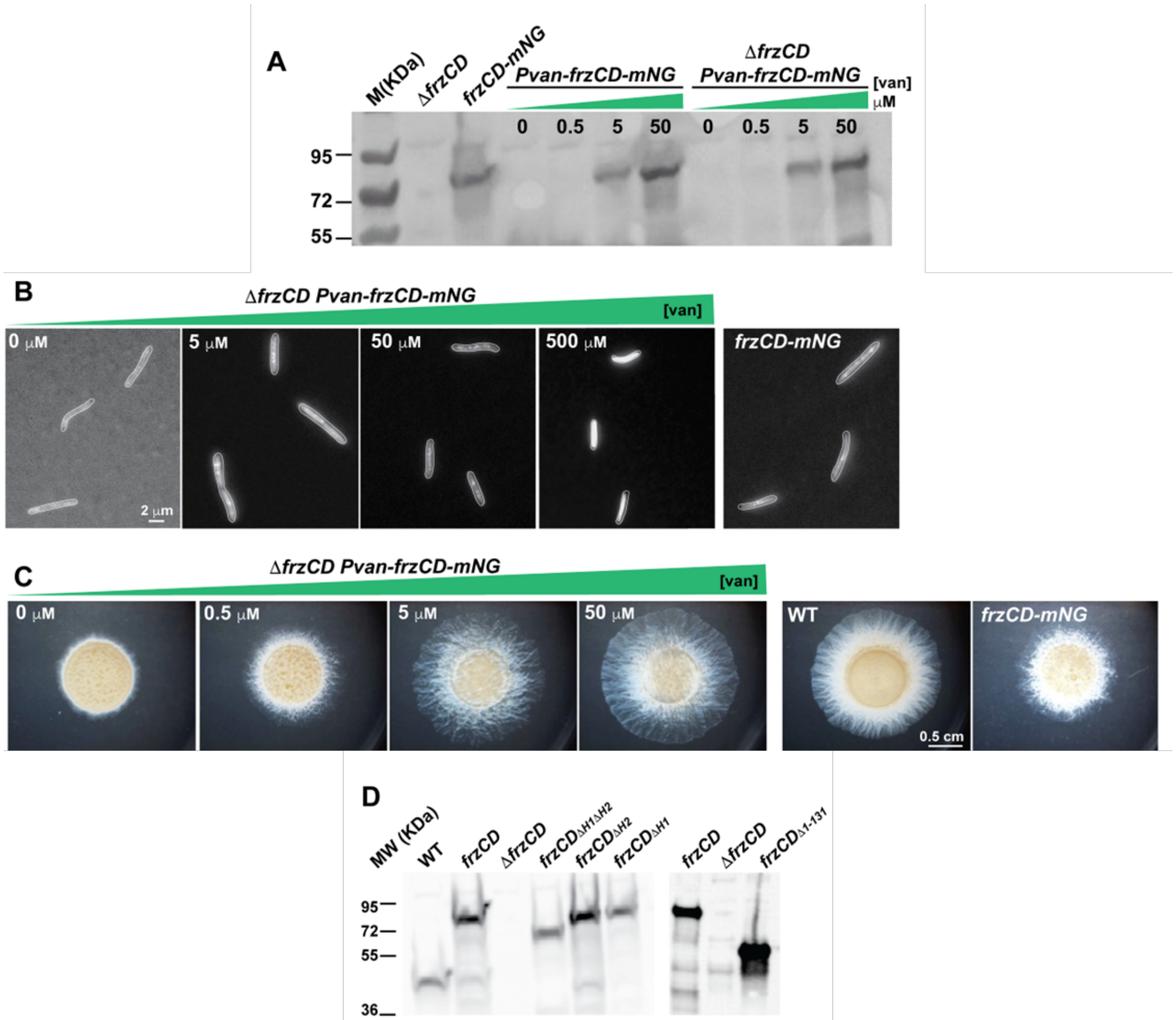

Supplementary Figure S3. FrzCD-mNG forms clusters at the nucleoid.

A. Representative Western blot with α-FrzCD antibodies on whole cell extracts of strains DZ2 (wild type), EM525 (Δ*frzCD*), EM724 (*frzCDmNG*) and EM885 (Δ*frzCD* P_{van}-*frzCDmNG*). P_{van}-*frzCDmNG* and Δ*frzCD* P_{van}-*frzCDmNG* were grown in the presence of the indicated concentrations of vanillate.

B. Δ*frzCD* P_{van}-*frzCDmNG* was grown in the presence of the indicated concentrations of vanillate and imaged using a fluorescence microscope.

C. The same cells were used for motility phenotypes on 0.5% CYE agar and imaged at 48h.

D. Western blot with α-FrzCD antibodies on cell extract of strains DZ2 (wild type), EM525 (Δ*frzCD*), EM885 (Δ*frzCD* P_{van}-*frzCDmNG*), EM914 (Δ*frzCD* P_{van}-*frzCDmNG*$^{\Delta H1}$), EM913 (Δ*frzCD* P_{van}-*frzCDmNG*$^{\Delta H2}$), EM911 (Δ*frzCD* P_{van}-*frzCDmNG*$^{\Delta H1\Delta H2}$) and EM908 (Δ*frzCD* P_{van}-*frzCDmNG*$^{\Delta 1\text{-}131}$).

Figure S4

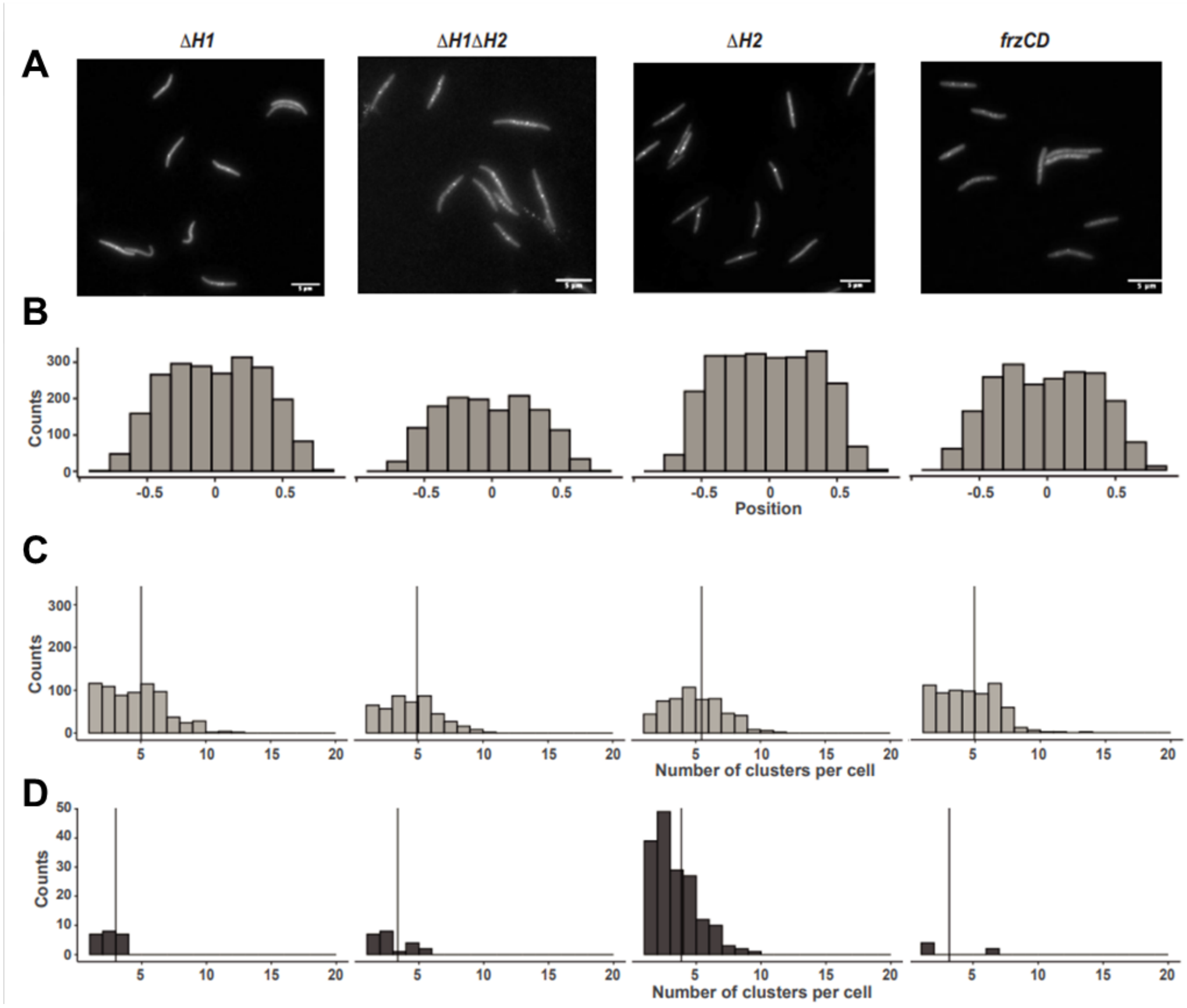

Supplementary Figure S4. Deletions of different HAMP domains have different effects on clusters number and intensity.

A. Large views of micrographs of *M. xanthus* cells from strains EM885 (Δ*frzCD* P_{van}-*frzCDmNG*), EM914 (Δ*frzCD* P_{van}-*frzCDmNG*$^{\Delta H1}$), EM913 (Δ*frzCD* P_{van}-*frzCDmNG*$^{\Delta H2}$) or EM911 (Δ*frzCD* P_{van}-*frzCDmNG*$^{\Delta H1\Delta H2}$). Scale bars correspond to 5 µm.

B. Absolute number of clusters as a function of their position in cells (from -1 to +1 on the y axis). "0" is the cell center; 0.5 and -0.5 are quarter positions.

C. Absolute number of low-fluorescence clusters per cell as a function of their fluorescence intensity (arbitrary units).

D. Absolute number of high-fluorescence clusters per cell as a function of their fluorescence intensity (arbitrary units).

Figure S5

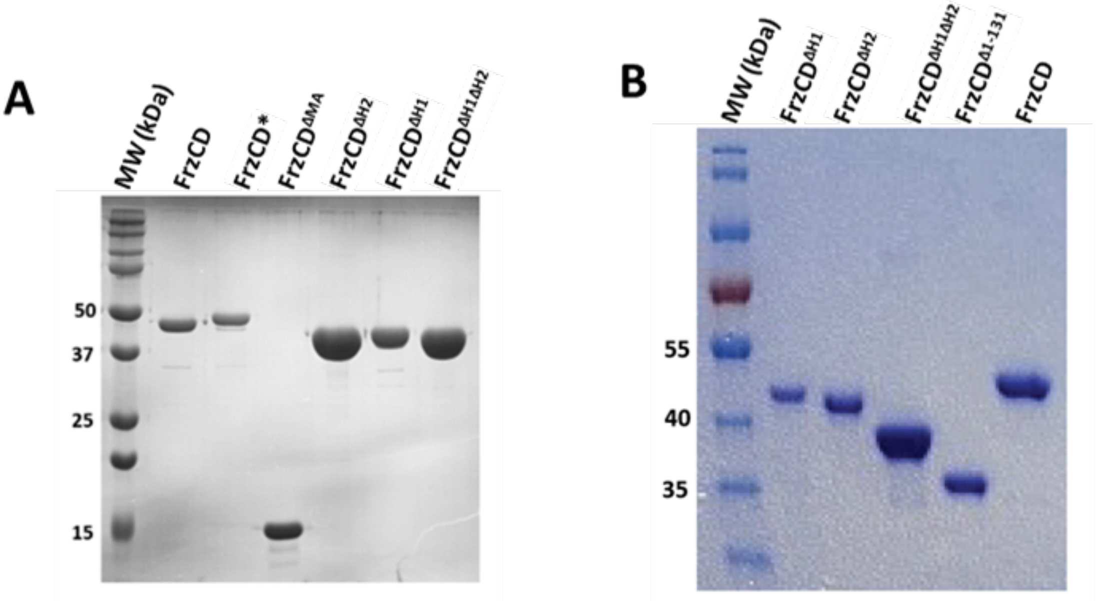

Supplementary Figure S5. The proteins used in this study are stably expressed.

A, B. 12 % **(A)** or 10 % **(B)** SDS-PAGE profiles of the purified FrzCD constructs used in this study. Proteins in (A) were used to perform the experiments shown on Figure 5A, Figure 6, and Supplementary Figure S6 and S7. Proteins in (B) were used to perform the experiments shown on Figures 5 (B-D).

Figure S6

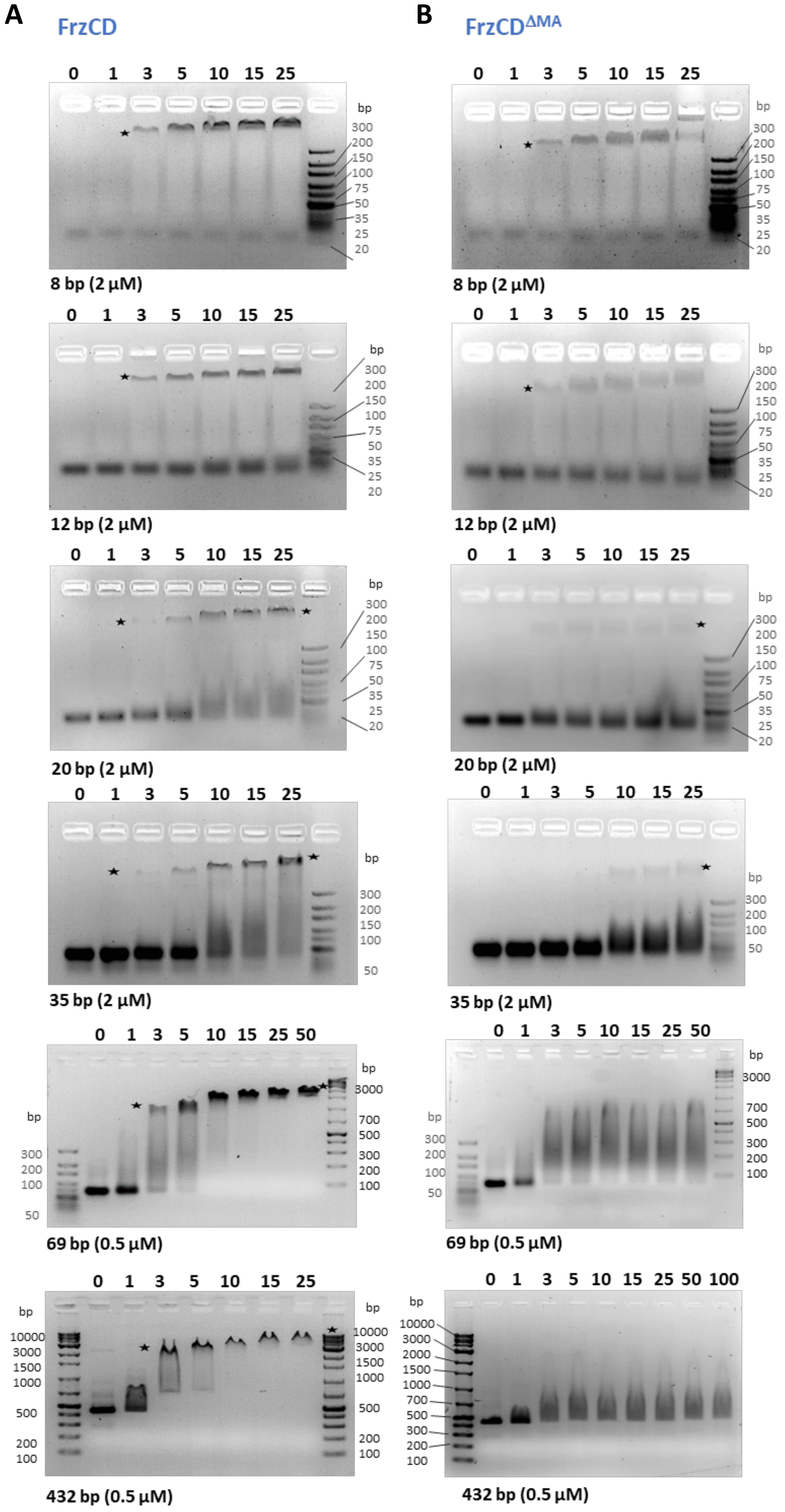

Supplementary Figure S6. MA domain stabilizes FrzCD binding to longer DNA fragments.

A-B. Representative electrophoretic mobility shift assays (EMSA) resolving the FrzCD and FrzCD$^{\Delta MA}$ binding to DNA. The protein concentrations of purified FrzCD protein domains are indicated corresponding to each lane and were incubated with the indicated concentration and length of DNA fragments from 8 to 432 bp (a star symbol in the gels marks supershifts).

Figure S7

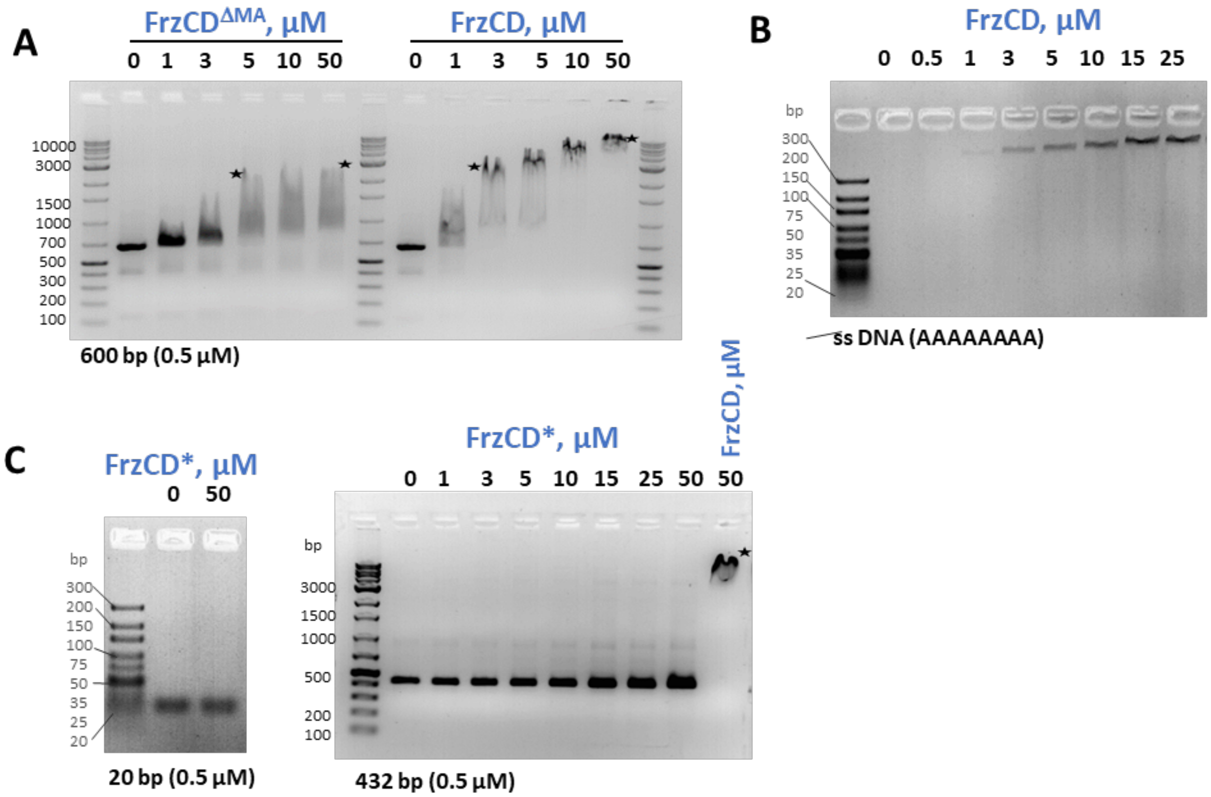

Supplementary Figure S7. Characteristics of FrzCD DNA binding

A. MA domain influences stable supershifts of longer DNA (binding profile comparison of 600-bp is shown; a star symbol in the gels marks supershifts).

B. FrzCD binds to single-stranded DNA. 1 µM of 8-base poly-A ssDNA titrated with increasing FrzCD concentration from 0 to 25 µM, according to lane labels.

C. FrzCD* shows no binding to DNA, and hence, no shift is observed in EMSA.

Supplementary Tables

Supplementary Table S1. Strains used in this study

M. xanthus	**Genotype**	**Reference or Source**
EM814	*DZ2*, Wild type	Campos and Zusman, 1975
EM525	Δ*frzCD*	Bustamante et al., 2004
EM724	*frzCD-mNeongreen*	This study
EM777	$frzCD^{\Delta H1}$*-mNeongreen*	This study
EM778	$frzCD^{\Delta H2}$*-mNeongreen*	This study
EM779	$frzCD^{\Delta H1H2}$*-mNeongreen*	This study
EM889	$frzCD^{\Delta H1}$	This study
EM775	$frzCD^{\Delta H2}$	This study
EM776	$frzCD^{\Delta H1H2}$	This study
EM886	P^{van}*-frzCD-mNeongreen*	This study
EM885	Δ*frzCD,* P^{van}*-frzCD-mNeongreen*	This study
EM914	Δ*frzCD,* P^{van}-$frzCD^{\Delta H1}$*-mNeongreen*	This study
EM913	Δ*frzCD,* P^{van}-$frzCD^{\Delta H2}$*-mNeongreen*	This study
EM911	Δ*frzCD,* P^{van}-$frzCD^{\Delta H1H2}$*-mNeongreen*	This study
EM1083	*pBJ114::frzF*	This study
EM1084	$frzCD^{\Delta H1}$*, pBJ114::frzF*	This study
EM1085	$frzCD^{\Delta H2}$*, pBJ114::frzF*	This study
EM1086	$frzCD^{\Delta H1H2}$*, pBJ114::frzF*	This study
E. coli	**Genotype**	**Reference or Source**
DH5α	*F- Φ80lacZΔM15 Δ(lacZYA-argF) U169 recA1 endA1 hsdR17 (rK-, mK+) phoA supE44 λ- thi-1 gyrA96 relA1*	New England Biolabs
BL21-AI	F^-*ompT* *hsd*S_B (r_B^- m_B^-) *gal dcm ara*B::T7RNAP-*tet*A	Invitrogen

Supplementary Table S2. Plasmids used in this study

Plasmid	Insert	Source
pKY480	Empty vector, containing *KanR* and *SucS* cassettes for generating mutants and insertions in *M. xanthus*	Mauriello et al., 2009
pEM517	pKY480 with *frzCD-mNeongreen*	This study
pBJ114	Empty vector containing *KanR* and *GalS* cassettes for generating mutants and insertions in *M. xanthus*	Bustamante et al., 2004
pEM566	pBJ114 with *frzCD*$^{\Delta H1}$	This study
pEM567	pBJ114 with *frzCD*$^{\Delta H2}$	This study
pEM569	pBJ114 with *frzCD*$^{\Delta H1 \quad \Delta H2}$	This study
pMR3690	Empty vector containing a KanR cassette and a promoter for inducible expression by vanillate in *M. xanthus*	Iniesta et al., 2012
pEM627	pMR3690 with *frzCD-mNeongreen*	This study
pEM646	pMR3690 with *frzCD*$^{\Delta H1}$*-mNeongreen*	This study
pEM643	pMR3690 with *frzCD*$^{\Delta H2}$*-mNeongreen*	This study
pEM644	pMR3690 with *frzCD*$^{\Delta H1H2}$*-mNeongreen*	This study
pEM706	pBJ114 with truncated *frzF*	This study
FrzCD	pHis17 with *frzCD*	This study
FrzCD$^{\Delta MA}$	pHis17 with *frzCD*$^{\Delta MA}$	This study
FrzCD$^{\Delta H1}$	pHis17 with *frzCD*$^{\Delta H1}$	This study
FrzCD$^{\Delta H2}$	pHis17 with *frzCD*$^{\Delta H2}$	This study
FrzCD$^{\Delta H1H2}$	pHis17 with *frzCD*$^{\Delta H1H2}$	This study
FrzCD*	pHis17 with *frzCD*$^{K9E\_K13E\_R15E\_R17E\_K18E}$	This study
pETphos	Empty vector for the synthesis of recombinant proteins with a N-terminal His-tag fusion, containing a very efficient TEV protease cleavage site and devoid of putative phosphoacceptors in the His-tag fusion. It derives from pET28.	Canova et al., 2008
pEM410	pETphos with *frzCD*	Moine et al., 2017
pEM414	pETphos with *frzCD*$^{\Delta 1\text{-}131}$	Moine et al., 2017
pEM663	pETphos with *frzCD*$^{\Delta H1}$	This study
pEM662	pETphos with *frzCD*$^{\Delta H2}$	This study
pEM658	pETphos with *frzCD*$^{\Delta H1H2}$	This study

Supplementary Table S3. Primers used for restriction-free cloning of domain-wise deletion constructs

Primer	Sequence (5' to 3')
$FrzCD^{K9E_K13E_R15E_R17E_K18E}$_f	CCCCCAACGAGGAGCCCGCTGGCGAGGCTGAAGCCGAGGAGGCCCCCGCCTCCGAGGCCGCGGCC
$FrzCD^{\Delta H1}$_f	GGCGCCACGAACGCGGCGTCGCGCAAGCATGTGGCGGCG
$FrzCD^{\Delta H1\Delta H2}$_f	GGCGCCACGAACGCGGCGTCGGTGCGGGAGATCAACGAG
$FrzCD^{\Delta H2}$_f	CAGTTCGCGGCCTCCGAGCACGTGCGGGAGATCAACGAG
$FrzCD^{\Delta MA}$_r	GCTTTTAATGATGATGATGATGATGGGATCCGAAGGTGCGCAGCGTCTCGATG

Supplementary Table S4. List of DNA sequences used for EMSA

DNA length	Sequence (5' to 3')
8bp	ACTGCAGT
ss DNA	AAAAAAAA
12 bp	CTCACTATAGGG
20 bp	TAATACGACTCACTATAGGG
35 bp	GTCACCTGCTCTAGCTAATAGACTGAGCCGAGGTG
69 bp	CTTGCAGTAGAGCTGACCATGATTACGCCATCAGCAGCTCCAGGTC GTACCTCCAGCTACCAATCCCCG
178bp	GGATCGCATCTGCCTACATCCCCAGCTCCCGGTCGGTCCGCTCGG AACCTAGCCCGGGTCAAAGACCCGGGGTCTATGTTGACCCATTTGG TGGGGATGGGTCTAATTGGACCTGGCGTTTTTTCGCGCCATGGAAA TGTCAAGGCCCGTGATTCCAGACTGTTGGGCAGGGAGTT
432 bp	TAATACGACTCACTATAGGGAGACCACAACGGTTTCCCTCTAGAAAT AATTTTGTTTAACTTTAAGAAGGAGATATACATATGTCCCTGGACACC CCCAACGAGAAGCCCGCTGGCAAGGCTCGCGCCCGGAAGGCCCC CGCCTCCAAGGCCGGCGCCACGAACGCGGCGTCGACCTCTTCCTC CACCAAGGCCATCACCGACACGCTGCTGACGGTGCTGTCCGGCAA CCTGCAGGCCCGCGTGCCCAAGGAGCTGGTCGGTGAGTCCGGCGT GGAGCTGGCGCACCTGCTCAACCAGGTGCTGGACCAGTTCGCGGC CTCCGAGCACCGCAAGCATGTGGCGGCGCAGGAGATCGACCAGGC GTTGGATGCGCTCATCGGCCTGGTGCGCGAGGGCGGATCCCATCA TCATCATCATCATTAAAAGC

Supplementary Table S5: Summary of the SEC-MALS profile peaks and their corresponding expected and estimated molecular masses.

Panel peak number	Probable Protein/DNA/Protein-DNA species	Theoretical Molar Mass (kDa)	Observed Molar Mass (kDa)
A	FrzCD dimer (89 kDa) +35-bp DNA (22 kDa)	111	111 ± 0.3
	FrzCD dimer (89 kDa)	89	89
	35-bp DNA (22 kDa)	22	22
B	FrzCD$^{\Delta MA}$ (31 kDa) + 35-bp DNA (22 kDa)	53	47 ± 2
C.1	35-bp DNA (22 kDa)	22	21.8 ± 0.3
C.2	FrzCD dimer (89 kDa)	89	85.8 ± 1.2
C.3	Higher order species		138.2 ± 1.2
C.4	Higher order species		199 ± 5
D.1	35-bp DNA (22 kDa)	22	24.7 ± 0.7
D.2	FrzCD$^{\Delta H2}$ (80 kDa) + 35-bp DNA (22 kDa)	102	107 ± 5.2
D.3	Higher-order species (broad peak)		294 ± 12
